# Supplementary material for: Reduced mortality but elevated venous thromboembolism risk following knee and hip arthroplasty in patients with rheumatoid arthritis: A general population-based cohort study
Source: PLoS One. 2025 Nov 7;20(11):e0335453. doi: 10.1371/journal.pone.0335453 (PMC12594356; doi:10.1371/journal.pone.0335453)
Supplement: S1 Table — (S1 Table.DOCX) [file pone.0335453.s001.docx]

**SUPPLEMENTARY MATERIALS**

**Title:** Reduced mortality and cardiovascular risk but elevated venous thromboembolism risk following knee and hip arthroplasty in patients with rheumatoid arthritis: a general population-based cohort study.

**Authors:** Xinjia Deng^1,2,3^, MD; Na Lu^4^, MPM; Dongxing Xie^1,2,3^, MD; Hui Li^1,2,3^, MD; Haochen Wang^1,2,3#^, MD

**Affiliations:**

1. Department of Orthopaedics, Xiangya Hospital, Central South University, Changsha, Hunan, China;
2. Key Laboratory of Aging-related Bone and Joint Diseases Prevention and Treatment, Ministry of Education, Xiangya Hospital, Central South University, Changsha, China;
3. Hunan Key Laboratory of Joint Degeneration and Injury, Changsha, Hunan, China;
4. Arthritis Research Canada, Richmond, BC, Canada.

**Correspondence to**: **Haochen Wang**, Department of Orthopaedics, Xiangya Hospital, Central South University, 87 Xiangya Road, Changsha, Hunan, China, 410008; E-mail: hausenwong@csu.edu.cn

**S1 Table. Baseline characteristics in the propensity score-matched cohort**

|  | **Knee** | | | **Hip** | | |
| --- | --- | --- | --- | --- | --- | --- |
|  | **KA** | **Non-KA** | **SMDs** | **HA** | **Non-HA** | **SMDs** |
| **Participants, n** | 2,387 | 2,387 |  | 1,681 | 1,681 |  |
| **Demographics** |  |  |  |  |  |  |
| Age, mean (SD), y | 66.4 (10.8) | 66.5 (12.0) | 0.015 | 68.1 (11.3) | 68.3 (11.9) | 0.015 |
| Male, % | 25.3 | 25 | 0.008 | 25.4 | 26.4 | 0.023 |
| Socioeconomic deprivation index score, mean (SD)^a^ | 2.7 (1.4) | 2.7 (1.4) | 0.003 | 2.7 (1.4) | 2.7 (1.4) | 0.026 |
| **RA duration, mean (SD)** | 12.8 (10.5) | 13.0 (11.2) | 0.022 | 12.5 (10.6) | 12.8 (11.0) | 0.032 |
| **Body mass index, mean (SD)** | 28.0 (5.8) | 28.1 (6.0) | 0.018 | 26.6 (5.3) | 26.6 (5.4) | 0.003 |
| **Smoking status, %** |  |  | 0.008 |  |  | 0.045 |
| None | 55.2 | 55.6 |  | 52.4 | 53.7 |  |
| Past | 32 | 31.8 |  | 32.9 | 33.1 |  |
| Current | 12.8 | 12.6 |  | 14.7 | 13.1 |  |
| **Drinking alcohol, %** |  |  | 0.027 |  |  | 0.035 |
| None | 26.9 | 27.8 |  | 29.1 | 28.7 |  |
| Past | 3.5 | 3.1 |  | 3.7 | 3.2 |  |
| Current | 69.6 | 69.1 |  | 67.1 | 68.1 |  |
| **Comorbidity, %** |  |  |  |  |  |  |
| CCI, mean (SD) | 0.8 (1.1) | 0.8 (1.1) | 0.05 | 0.9 (1.1) | 0.8 (1.2) | 0.045 |
| Myocardial infarction | 4.3 | 5.2 | 0.043 | 5.5 | 5.5 | 0.003 |
| Atrial fibrillation | 4.5 | 4.2 | 0.012 | 4.6 | 4 | 0.029 |
| Ischaemic heart disease | 11.8 | 12.5 | 0.022 | 14.8 | 14.5 | 0.008 |
| Peripheral vascular disease | 1.2 | 1.3 | 0.011 | 2 | 1.7 | 0.018 |
| Congestive heart failure | 2.4 | 2.4 | <0.001 | 3.5 | 3.5 | <0.001 |
| Valvular heart disease | 1.8 | 1.9 | 0.006 | 3.2 | 3.1 | 0.003 |
| Transient ischaemic attack | 2.8 | 2.8 | 0.003 | 3.1 | 2.9 | 0.010 |
| Angina | 7.1 | 7.7 | 0.022 | 9.2 | 9.3 | 0.004 |
| Other circulatory diseases | 31.7 | 31.8 | 0.001 | 31.1 | 31.5 | 0.010 |
| Stroke | 2.8 | 2.1 | 0.046 | 4.1 | 3.6 | 0.025 |
| Hypertension | 48.4 | 48.6 | 0.003 | 48.2 | 47.4 | 0.015 |
| COPD | 4.9 | 4.8 | 0.008 | 7.6 | 7.3 | 0.009 |
| Chronic kidney disease | 10.7 | 10.2 | 0.016 | 13.3 | 13.3 | 0.002 |
| Liver disease | 4.2 | 4.2 | <0.001 | 3.5 | 3.4 | 0.007 |
| Diabetes | 11.2 | 11.4 | 0.007 | 11.4 | 11.7 | 0.009 |
| Cancer | 10.3 | 13.7 | 0.106 | 10.9 | 13.5 | 0.080 |
| Pneumonia or other infections | 8.7 | 8.6 | 0.001 | 9 | 8.7 | 0.010 |
| Depression | 13.2 | 13.4 | 0.006 | 13.1 | 12.1 | 0.029 |
| Venous thromboembolism | 5.9 | 4.5 | 0.062 | 6.9 | 5.5 | 0.059 |
| Varicose veins | 11.3 | 11 | 0.009 | 11.2 | 12.8 | 0.051 |
| Other inflammatory conditions | 15.4 | 15.4 | <0.001 | 16.5 | 16.8 | 0.008 |
| High fall risk | 13.8 | 13.8 | 0.001 | 16 | 17.4 | 0.038 |
| Hip fracture | 2.2 | 2.3 | 0.008 | 10.1 | 9.8 | 0.010 |
| Hyperlipidaemia | 11.5 | 11.9 | 0.012 | 11.9 | 12.6 | 0.020 |
| Dementia | 0.3 | 0.4 | 0.007 | 0.8 | 1 | 0.025 |
| Osteoporosis | 20.7 | 21.3 | 0.014 | 25 | 25.2 | 0.003 |
| Seizure | 0.3 | 0.3 | <0.001 | 0.5 | 0.7 | 0.023 |
| Trauma | 1.3 | 1.4 | 0.011 | 1.4 | 1.6 | 0.015 |
| Peptic ulcer | 8.4 | 8.8 | 0.012 | 10.5 | 10.3 | 0.006 |
| GERD | 15.2 | 15.2 | 0.001 | 15.3 | 15.7 | 0.010 |
| Gastritis | 28 | 28.7 | 0.017 | 24.8 | 26.1 | 0.029 |
| **Medication, %** |  |  |  |  |  |  |
| Nitrates | 4.5 | 5.2 | 0.035 | 5.9 | 5.2 | 0.029 |
| Antihypertensive medicine | 55.8 | 55.7 | 0.003 | 58.5 | 57.8 | 0.016 |
| ACE inhibitors | 21.8 | 21.6 | 0.005 | 22.4 | 21.7 | 0.016 |
| β-Blockers | 19.6 | 20.6 | 0.023 | 20.6 | 20.5 | 0.004 |
| Calcium channel blockers | 21.2 | 21.1 | 0.003 | 21.6 | 22 | 0.010 |
| Angiotensin receptor blockers^b^ | 9.7 | 9.7 | <0.001 | 8.9 | 9.9 | 0.037 |
| Statin | 26.6 | 27.9 | 0.029 | 28.7 | 28.7 | 0.001 |
| Benzodiazepines | 13 | 13.4 | 0.014 | 15.6 | 14.8 | 0.025 |
| SSRI | 10.9 | 11.7 | 0.028 | 11.2 | 11.1 | 0.002 |
| SNRI | 1.6 | 1.6 | 0.003 | 2 | 2 | <0.001 |
| Aspirin | 19.3 | 19.6 | 0.007 | 22.1 | 22.1 | 0.001 |
| Loop diuretics | 13.2 | 13.6 | 0.014 | 15.6 | 15.6 | <0.001 |
| Thiazide-like diuretic | 17.3 | 16.4 | 0.023 | 17.2 | 16.8 | 0.009 |
| Potassium sparing diuretics | 3.6 | 3.7 | 0.004 | 4 | 4 | 0.003 |
| DMARDs | 61.6 | 61 | 0.014 | 59.1 | 57.1 | 0.041 |
| Azathioprine | 1.8 | 1.7 | 0.006 | 2.9 | 3 | 0.007 |
| Hydroxychloroquine | 11.4 | 11.2 | 0.005 | 11.5 | 10.9 | 0.017 |
| Methotrexate | 6.2 | 5.7 | 0.021 | 5.1 | 4.8 | 0.016 |
| Leflunomide | 38.6 | 39.5 | 0.017 | 37.7 | 35.1 | 0.054 |
| Sulfasalazine | 20.1 | 19.9 | 0.005 | 17.3 | 18.3 | 0.026 |
| Mycophenolate | 0.2 | 0.3 | 0.017 | 0.1 | 0.2 | 0.028 |
| Sodium Aurothiomalate | 1.8 | 1.8 | <0.001 | 1.3 | 1.2 | 0.011 |
| Biologics | 0.3 | 0.2 | 0.018 | 0.2 | 0.1 | 0.028 |
| Glucocorticoids | 33.7 | 32.6 | 0.023 | 33 | 32.8 | 0.004 |
| Anticoagulant | 4.7 | 4.9 | 0.01 | 5.2 | 5.3 | 0.005 |
| Opioids | 41.5 | 41.1 | 0.008 | 47.2 | 48.3 | 0.023 |
| PPI | 52.1 | 53 | 0.018 | 52.3 | 52.9 | 0.011 |
| Bisphosphonates | 21.7 | 22.7 | 0.026 | 24.1 | 23.7 | 0.010 |
| Antidiabetics | 6.5 | 6.6 | 0.005 | 6.7 | 6.6 | 0.005 |
| NSAIDs | 76.6 | 76.4 | 0.006 | 74.9 | 74.4 | 0.011 |
| **Health care utilization, mean (SD)** |  |  |  |  |  |  |
| General practice visits | 17.2 (15.3) | 17.1 (15.2) | <0.001 | 17.6 (16.2) | 17.3 (14.9) | 0.017 |
| Hospitalizations | 1.2 (2.0) | 1.3 (1.8) | 0.025 | 1.4 (2.2) | 1.4 (2.2) | 0.002 |
| Specialist referrals | 1.4 (1.9) | 1.5 (1.9) | 0.011 | 1.5 (2.1) | 1.5 (2.1) | 0.002 |

^a^ Socioeconomic Deprivation Index Score was measured by the Townsend Deprivation Index, which was grouped into quintiles from 1 (least deprived) to 5 (most deprived).

^b^ All angiotensin receptor blockers were included other than losartan.

KA, knee arthroplasty; HA, hip arthroplasty; SMDs, standardized mean differences; RA, rheumatoid arthritis; ACE, angiotensin-converting enzyme; NSAID, nonsteroidal anti-inflammatory drug; SNRI, serotonin–norepinephrine reuptake inhibitor; SSRI, selective serotonin reuptake inhibitor; DMARDs, disease-modifying antirheumatic drugs; PPI, proton pump inhibitor; GERD, Gastroesophageal reflux disease; CCI, Charlson Comorbidity Index; COPD, Chronic obstructive pulmonary disease.
