# Supplementary material for: Reduced mortality but elevated venous thromboembolism risk following knee and hip arthroplasty in patients with rheumatoid arthritis: A general population-based cohort study
Source: PLoS One. 2025 Nov 7;20(11):e0335453. doi: 10.1371/journal.pone.0335453 (PMC12594356; doi:10.1371/journal.pone.0335453)
Supplement: Attachment — Submitted filename: Response to Reviewers.docx [file pone.0335453.s003.docx]

**Editors’ Comments to the Authors:**

After careful consideration, we feel that it has merit but does not fully meet PLOS ONE’s publication criteria as it currently stands. Therefore, we invite you to submit a revised version of the manuscript that addresses the points raised during the review process.

The reviewers who assessed your manuscript have suggested changes and expressed concerns, which you can find in the attached document. I kindly ask you to evaluate the suggested changes and respond to the questions raised in order to improve the text and enhance the publishability of your work.

**Response:** We appreciate the editor’s comment. We provide a point-by-point response to the reviewers’ comments. We hope that our responses are satisfactory and that the changes we have made in the text reflect our responsiveness to the comments and suggestions.

**Reviewer #1:**

Comments to the Author

The manuscript is a valuable contribution to the field of orthopaedic surgery and rheumatoid arthritis research. The statistical approach is sound, and the conclusions are well-supported by the data. I commend the authors for their strong work.

**Response:** We are very grateful to the reviewer for the valuable comments and suggestions that have helped improve both the content and presentation of our work. Per the reviewer’s suggestions, the manuscript has been reviewed and reorganized.

**Comment 1**

Abstract

The key findings are well summarized, but the risk estimates for CVD are not statistically significant. Consider emphasizing that the trend suggests a reduction in risk but is not conclusive.

**Response:** We appreciate the reviewer’s suggestions. Per the reviewer’s suggestion, we have modified the description of findings in the **Abstract** section to emphasize that the trend suggests a reduction in risk but is not conclusive.

**Action:** “Our population-based cohort study provides the first evidence that knee and hip arthroplasty are associated with a lower risk of mortality and a trend toward reduced CVD risk but an increased risk of VTE among patients with RA.” **(Page 3, line 52-55, in the clean copy of the revised manuscript)**

**Comment 2** The sample size should be reiterated clearly to highlight the study’s strength.

**Response:** Many thanks for the reviewer’s suggestion. We have added the description of the sample size on all-cause mortality, cardiovascular disease (CVD), and venous thromboembolism (VTE) in the **Abstract** section.

**Action:** “The primary outcome was all-cause mortality (n=4,774 for knee arthroplasty, n=3,362 for hip arthroplasty). The secondary outcomes included incident CVD (n=4,350 for knee arthroplasty, n=2,390 for hip arthroplasty) and incident VTE (n=4,574 for knee arthroplasty, n=3,174 for hip arthroplasty).” **(Page 2, line 34-37, in the clean copy of the revised manuscript)**

**Comment 3**

Introduction

The background is well-written, but explicitly defining the study’s hypothesis earlier would improve readability.

Line 26: “Arthroplasty surgery is indicated for rheumatoid arthritis (RA) patients…” →Consider revising to “Arthroplasty is indicated for patients with rheumatoid arthritis (RA)...” (avoid “RA patients” for better readability).

**Response:** We appreciate the reviewer’s comments. Per the reviewer’s comment, we have revised the wording of this sentence in accordance with your suggestion in the **Abstract** section of the manuscript.

**Action:** “Arthroplasty is indicated for patients with rheumatoid arthritis (RA) who experience significant joint damage, including bone erosions, cartilage degradation and joint deformities.” **(Page 2, line 27-29, in the clean copy of the revised manuscript)**

**Comment 4**

Consider adding a brief statement acknowledging previous research that found no survival benefit to provide better contrast with the study’s findings.

**Response:** We appreciate the reviewer’s comment. Previous literature has suggested that patients with RA undergoing arthroplasty may present with comorbidities such as infections and osteoporosis ^1,2^, while no studies have yet investigated the survival benefits of arthroplasty among patients with RA. We have revised the corresponding content in the **Introduction** section.

References:

1	Ravi, B. et al. A systematic review and meta-analysis comparing complications following total joint arthroplasty for rheumatoid arthritis versus for osteoarthritis. Arthritis Rheum 64, 3839-3849, doi:10.1002/art.37690 (2012).

2	Xiao, P. L. et al. Prevalence and treatment rate of osteoporosis in patients undergoing total knee and hip arthroplasty: a systematic review and meta-analysis. Arch Osteoporos 17, 16, doi:10.1007/s11657-021-01055-9 (2022).

**Action:** “However, given multiple joint involvement, systemic comorbidities (including CVD, VTE, infections, postoperative joint dislocation, and osteoporosis) and polypharmacy, the benefits of these surgeries in terms of quality of life are not always clear among patients with RA ^12,15-17^.” **(Page 4, line 82-85, in the clean copy of the revised manuscript)**

References:

12	Burn, E. et al. The effect of rheumatoid arthritis on patient-reported outcomes following knee and hip replacement: evidence from routinely collected data. Rheumatology (Oxford) 58, 1016-1024, doi:10.1093/rheumatology/key409 (2019).

15	Momohara, S. et al. Efficacy of total joint arthroplasty in patients with established rheumatoid arthritis: improved longitudinal effects on disease activity but not on health-related quality of life. Mod Rheumatol 21, 476-481, doi:10.1007/s10165-011-0432-9 (2011).

16	Ravi, B. et al. A systematic review and meta-analysis comparing complications following total joint arthroplasty for rheumatoid arthritis versus for osteoarthritis. Arthritis Rheum 64, 3839-3849, doi:10.1002/art.37690 (2012).

17	Xiao, P. L. et al. Prevalence and treatment rate of osteoporosis in patients undergoing total knee and hip arthroplasty: a systematic review and meta-analysis. Arch Osteoporos 17, 16, doi:10.1007/s11657-021-01055-9 (2022).

**Comment 5**

Methods

Line 104: Clarify how loss to follow-up was handled. Were these patients censored, and if so, at what time point?

**Response:** We appreciate the reviewer’s suggestion. The loss to follow-up will be treated as censored data, an approach that has been documented in the statistical analysis section. The censoring time for these participants will be determined as the interval from baseline to their last recorded general practitioner (GP) visit date prior to the study endpoint (December 31, 2018).

**Comment 6** Line 133-140: The matching process could be better explained, particularly how greedy matching was implemented (e.g., caliper width used).

**Response:** We sincerely appreciate the reviewer’s insightful comment. The greedy matching algorithm, implemented through the SAS macro, utilizes a hierarchical digit-based approach to propensity score matching, which inherently defines caliper widths by adjusting precision levels. The specified caliper width is 0.1. We have added the corresponding content in the **Methods** section.

**Action:** “The greedy matching algorithm, implemented through the SAS macro, utilizes a hierarchical digit-based approach to propensity score matching, which inherently defines caliper widths by adjusting precision levels. The specified caliper width is 0.1.” **(Page 8, line 164-167, in the clean copy of the revised manuscript)**

**Comment 7**

Results

Line 197: Consider reporting absolute risk reduction (ARR) along with HRs to aid clinical interpretation.

**Response:** We appreciate the reviewer’s suggestion. Per the reviewer’s suggestion, we have reported absolute risk reduction (ARR) in the **Results** section.

**Action:** “Participants with knee arthroplasty had a 23% lower risk of all-cause mortality compared with those without knee arthroplasty (HR=0.77, 95% CI 0.65 to 0.90; ARR=1.71%) (**Table 1**; **Fig 1A**). We identified 366 deaths (40.6 per 1,000 person-years) in subjects with hip arthroplasty and 395 deaths in those without hip arthroplasty (43.9 per 1,000 person-years) during follow-up. The mean follow-up period was 5.4 years for the hip arthroplasty group and 5.4 years for the non-hip arthroplasty group. The rate of all-cause mortality among subjects with hip arthroplasty was lower, albeit non-statistically significant, than those without hip arthroplasty (HR=0.87, 95% CI 0.73 to 1.04; ARR=1.49%) (**Table 1**; **Fig 1B**).” **(Page 10, line 219-227, in the clean copy of the revised manuscript)**

“Participants with knee arthroplasty had a 14% lower, albeit non-statistically significant, risk of CVD than their counterparts (HR=0.86, 95% CI 0.73 to 1.01; ARR=0.09%) (**Table 2**; **Fig 2A**). During the follow-up period, 135 cases of CVD in the hip arthroplasty group and 146 CVD cases in the comparison group were identified. Incidence rates per 1,000 person-years for the hip arthroplasty and comparison cohorts were 17.6 and 19.6, respectively. The mean follow-up period was 5.2 years for the hip arthroplasty group and 5.1 years for the non-hip arthroplasty group. The risk of CVD, albeit non-statistically significant, was lower in subjects with hip arthroplasty than those without hip arthroplasty (HR=0.84, 95% CI 0.69 to 1.02; ARR=0.77%) (**Table 2**; **Fig 2B**).” **(Page 12, line 249-258, in the clean copy of the revised manuscript)**

“Compared with those without knee arthroplasty, the HR of VTE for subjects with knee arthroplasty was 1.63 (95% CI 1.23 to 2.17) and the corresponding ARR was 0.97% (**Table 3**; **Fig 3A**). Similar results were also observed when the association between hip arthroplasty and the risk of VTE was examined (7.98 vs. 3.88 per 1,000 person-years). The mean follow-up period was 5.3 years for the hip arthroplasty group and 5.2 years for the non-hip arthroplasty group. The corresponding HR was 2.19 (95% CI 1.54 to 3.11) and ARR was 2.14% (**Table 3**; **Fig 3B**).” **(Page 14, line 279-285, in the clean copy of the revised manuscript)**

**Comment 8** Tables 1-3: Ensure consistency in presenting statistical significance (bold or asterisk for p < 0.05).

**Response:** We are thankful for the reviewer’s feedback and the statistically significant results in Tables 1-3 have been bolded for emphasis.

**Action:** We have revised **Table 1-3**. **(Page 10-15, in the clean copy of the revised manuscript)**

**Comment 9** Table 1 footnotes: Ensure uniform formatting for hazard ratios (HR) and confidence intervals (CI).

**Response:** Many thanks for the reviewer’s comment. We have revised the footnotes in Table 1 to ensure uniform formatting.

**Action:** We have revised **Table 1** footnotes. **(Page 11, line 233-234, in the clean copy of the revised manuscript)**

**Comment 10**

Discussion

Line 275: “Our findings also showed that either knee or hip arthroplasty decreased the risk of CVD and increased the risk of VTE...” → Consider specifying the magnitude of risk reduction/increase directly in the sentence.

**Response:** We acknowledge the reviewer’s suggestion with appreciation. We have added the corresponding content to the **Discussion** section according to your suggestion.

**Action:** “Our findings showed a substantially lower risk of all-cause mortality (23% for KA, 13% for HA) in patients with RA after knee or hip arthroplasty, indicating significant protective associations for both KA and HA regarding all-cause mortality. Our study revealed that both KA and HA demonstrated cardioprotective properties in patients with RA, exhibiting a trend toward reduced cardiovascular disease risk (14% for KA, 16% for HA). However, this potential cardiovascular benefit must be weighed against a substantial elevation in VTE risk, with postoperative analyses showing 63% and 119% increased incidence rates for KA and HA recipients respectively.” **(Page 15-16, line 305-313, in the clean copy of the revised manuscript)**

**Comment 11** Line 279: The discussion on mechanisms is strong but should also consider alternative explanations (e.g., RA patients undergoing arthroplasty may have better baseline health or access to care).

**Response:** We are thankful for the reviewer’s input. Per the reviewer’s comment, we have added the alternative explanations in the **Abstract** section.

**Action:** “Third, patients with RA undergoing arthroplasty may inherently have better baseline health or access to healthcare. Fourth, arthroplasty may attenuate systemic inflammation, a key driver of CVD in RA. Chronic inflammation, marked by elevated cytokines (e.g., IL-6, TNF-α) and acute-phase reactants like CRP, accelerates endothelial dysfunction and plaque instability ^27,60^. By alleviating localized joint inflammation and reducing inflammatory burden, arthroplasty may lower inflammatory burden, indirectly improving cardiovascular outcomes. Fifth, perioperative multidisciplinary care optimizes cardiovascular risk factors. Preoperative optimization of cardiac function, intraoperative hemodynamic monitoring, and postoperative rehabilitation protocols may mitigate surgical stress and enhance long-term outcomes ^61^.” **(Page 17, line 345-355, in the clean copy of the revised manuscript)**

References:

27	England, B. R., Thiele, G. M., Anderson, D. R. & Mikuls, T. R. Increased cardiovascular risk in rheumatoid arthritis: mechanisms and implications. BMJ 361, k1036, doi:10.1136/bmj.k1036 (2018).

60	Blum, A. & Adawi, M. Rheumatoid arthritis (RA) and cardiovascular disease. Autoimmunity reviews 18, 679-690, doi:10.1016/j.autrev.2019.05.005 (2019).

61	Heckert, S. L. et al. Long-term mortality in treated-to-target RA and UA: results of the BeSt and IMPROVED cohort. Annals of the rheumatic diseases 83, 161-168, doi:10.1136/ard-2023-224814 (2024).

**Comment 12** Line 312: The authors should acknowledge potential unmeasured confounders, such as biologic DMARD use and socioeconomic factors.

**Response:** We appreciate the reviewer’s suggestion. We have included a discussion of potential unmeasured confounders, such as biologic DMARD use in the **Discussion** section of the manuscript to acknowledge this limitation. The socioeconomic factors, particularly socioeconomic status (Townsend Deprivation Index), were systematically adjusted for as potential confounders. We recognize that residual confounding may persist despite these methodological precautions, and would be happy to incorporate any additional specific suggestions the reviewer might have for further strengthening this aspect of our analysis.

**Action:** “Third, the current data set does not contain information on the period of biological disease-modifying anti-rheumatic drugs (DMARDs) administration. Since studies have shown that biological DMARDs use was associated a decreased risk of mortality and cardiovascular events in patients with RA ^64,65^, without adjustment of biological DMARD could also introduce potential residual confounding.” **(Page 19, line 385-390, in the clean copy of the revised manuscript)**

References:

64	Rodriguez-Rodriguez, L. et al. Treatment in rheumatoid arthritis and mortality risk in clinical practice: the role of biologic agents. Clin Exp Rheumatol 34, 1026-1032 (2016).

65	Greenberg, J. D. et al. Tumour necrosis factor antagonist use and associated risk reduction of cardiovascular events among patients with rheumatoid arthritis. Ann Rheum Dis 70, 576-582 (2011).

**Comment 13** Line 374: The statement “coordinated care involving medicine, surgery, anaesthesia, and nursing throughout the perioperative process...” is important. Consider referencing specific perioperative guidelines for RA patients.

**Response:** We are thankful for the reviewer’s comment. Per the reviewer’s comment, we have added the corresponding reference regarding specific perioperative guidelines for RA patients to the **Discussion** section.

**Action:** “Therefore, it is essential for patients with RA who require surgery to have coordinated care involving medicine, surgery, anaesthesia, and nursing throughout the perioperative process, which facilitates the creation of a comprehensive interdisciplinary care plan before the operation ^11,69,84^.” **(Page 22, line 472-475, in the clean copy of the revised manuscript)**

“**REFERENCES**

11	Goodman, S. M. et al. 2017 American College of Rheumatology/American Association of Hip and Knee Surgeons Guideline for the Perioperative Management of Antirheumatic Medication in Patients With Rheumatic Diseases Undergoing Elective Total Hip or Total Knee Arthroplasty. Arthritis Rheumatol 69, 1538-1551, doi:10.1002/art.40149 (2017).” **(Page 26, line 544-547, in the clean copy of the revised manuscript)**

“**REFERENCES**

69	Akkara Veetil, B. M. & Bongartz, T. Perioperative care for patients with rheumatic diseases. Nat Rev Rheumatol 8, 32-41, doi:10.1038/nrrheum.2011.171 (2011).” **(Page 30, line 702-703, in the clean copy of the revised manuscript)**

“**REFERENCES**

84	Krause, M. L. & Matteson, E. L. Perioperative management of the patient with rheumatoid arthritis. World J Orthop 5, 283-291, doi:10.5312/wjo.v5.i3.283 (2014).” **(Page 31, line 742-743, in the clean copy of the revised manuscript)**

**Comment 14**

Conclusion

The conclusion is appropriate but could briefly emphasize clinical implications (e.g., “These findings suggest that arthroplasty may be considered not only for joint function but also for potential survival and cardiovascular benefits in carefully selected RA patients.”).

**Response:** We appreciate the reviewer’s comment and have added the corresponding content to the **Introduction** section.

**Action:** “Our population-based cohort study provides the first evidence that knee and hip arthroplasty are associated with a lower risk of mortality and a trend toward reduced CVD risk but an increased risk of VTE among patients with RA. These findings suggest that arthroplasty may be considered not only for joint function but also for potential survival and cardiovascular benefits in carefully selected patients with RA.” **(Page 22-23, line 478-482, in the clean copy of the revised manuscript)**

**Reviewer #2:**

**Comment 1**

Title

It did not emphasize the highlights of the study.

**Response:** We appreciate the reviewer’s comment. The title has been modified to emphasize the highlights of our study.

**Action:** “Reduced mortality and cardiovascular risk but elevated venous thromboembolism risk following knee and hip arthroplasty in patients with rheumatoid arthritis: a general population-based cohort study.” **(Page 1, line 1-3, in the clean copy of the revised manuscript)**

**Comment 2** The terms “survival benefits” were non-specific. It can be improved.

**Response:** Many thanks for the reviewer’s comment. We have revised the terms “survival benefits” to a more clinically precise terminology in accordance with standardized outcome definitions.

**Action:** “Reduced mortality and cardiovascular risk but elevated venous thromboembolism risk following knee and hip arthroplasty in patients with rheumatoid arthritis: a general population-based cohort study.” **(Page 1, line 1-3, in the clean copy of the revised manuscript)**

**Comment 3**

Abstract

Row 27-What it means “significant joint damage”?

**Response:** We appreciate the reviewer’s suggestion. In RA, significant joint damage involves bone erosions, cartilage degradation, joint deformities (e.g., ulnar deviation, swan-neck/boutonnière deformities), subluxation, and synovial proliferation. We have added the corresponding content to the **Abstract** section.

**Action:** “Arthroplasty is indicated for patients with rheumatoid arthritis (RA) who experience significant joint damage, including bone erosions, cartilage degradation and joint deformities.” **(Page 2, line 27-29, in the clean copy of the revised manuscript)**

**Comment 4** Row 29-Which type of “risk” was evaluated?

**Response:** We value the reviewer’s comment. The “risk” refers to “relative risk”. Per the reviewer’s comment, we have modified the manuscript accordingly.

**Action:** “Our aim was to evaluate the relation of knee arthroplasty or hip arthroplasty to all-cause mortality, relative risk of cardiovascular disease (CVD) and incident venous thromboembolism (VTE) among patients with RA.” **(Page 2, line 30-32, in the clean copy of the revised manuscript)**

**Comment 5**

Introduction

Row 56- the “increased risks” could be written with numbers and percentages.

**Response:** We appreciate the reviewer’s comment and have added the corresponding content to the **Introduction** section.

**Action:** “It is associated with increased risks of cardiovascular disease (CVD, 48%, pooled risk ratio=1.48 (95% CI 1.36 to 1.62)) ^2,3^, venous thromboembolism (VTE, 100%, adjusted hazard ratio=2.0 (95% CI, 1.9-2.2)) ^4,5^, and mortality (43%, HR=1.43 (95% CI 1.28 to 1.59)) ^6,7^.” **(Page 4, line 63-66, in the clean copy of the revised manuscript)**

References:

2	Avina-Zubieta, J. A., Thomas, J., Sadatsafavi, M., Lehman, A. J. & Lacaille, D. Risk of incident cardiovascular events in patients with rheumatoid arthritis: a meta-analysis of observational studies. Annals of the rheumatic diseases 71, 1524-1529, doi:10.1136/annrheumdis-2011-200726 (2012).

3	Kitas, G. D. & Gabriel, S. E. Cardiovascular disease in rheumatoid arthritis: state of the art and future perspectives. Annals of the rheumatic diseases 70, 8-14, doi:10.1136/ard.2010.142133 (2011).

4	Holmqvist, M. E. et al. Risk of venous thromboembolism in patients with rheumatoid arthritis and association with disease duration and hospitalization. Jama 308, 1350-1356, doi:10.1001/2012.jama.11741 (2012).

5	Zoller, B., Li, X., Sundquist, J. & Sundquist, K. Risk of pulmonary embolism in patients with autoimmune disorders: a nationwide follow-up study from Sweden. Lancet 379, 244-249, doi:10.1016/S0140-6736(11)61306-8 (2012).

6	Holmqvist, M., Ljung, L. & Askling, J. Mortality following new-onset Rheumatoid Arthritis: has modern Rheumatology had an impact? Ann Rheum Dis 77, 85-91, doi:10.1136/annrheumdis-2017-212131 (2018).

7	Solomon, D. H. et al. Cardiovascular morbidity and mortality in women diagnosed with rheumatoid arthritis. Circulation 107, 1303-1307, doi:10.1161/01.cir.0000054612.26458.b2 (2003).

**Comment 6** It is lacking information about advantages and disadvantages related with TKA and THA in RA patients. It can be improved.

**Response:** Many thanks for the reviewer’s suggestion. In accordance with the reviewer’s recommendation, we have added the corresponding content to the **Introduction** section.

**Action:** “The primary objective of knee and hip arthroplasty in patients with RA is to address the structural joint abnormalities, leading to significant improvements in pain and function ^12,13^. However, elevated risks of infections and complications like prosthetic loosening persist due to chronic inflammation and immunosuppressive therapies ^11^.” **(Page 4, line 74-78, in the clean copy of the revised manuscript)**

References:

11	Goodman, S. M. et al. 2017 American College of Rheumatology/American Association of Hip and Knee Surgeons Guideline for the Perioperative Management of Antirheumatic Medication in Patients With Rheumatic Diseases Undergoing Elective Total Hip or Total Knee Arthroplasty. Arthritis & rheumatology (Hoboken, N.J.) 69, 1538-1551, doi:10.1002/art.40149 (2017).

12	Burn, E. et al. The effect of rheumatoid arthritis on patient-reported outcomes following knee and hip replacement: evidence from routinely collected data. Rheumatology (Oxford) 58, 1016-1024, doi:10.1093/rheumatology/key409 (2019).

13	Harris, W. H. & Sledge, C. B. Total hip and total knee replacement (1). N Engl J Med 323, 725-731, doi:10.1056/NEJM199009133231106 (1990).

**Comment 7** The common complications related with TKA and THA in RA were not written, mainly VTE and CVD. It can be improved.

**Response:** Many thanks for the reviewer’s comment. Following the reviewer’s advice, we have added the common complications related with TKA and THA in RA to the **Introduction** section.

**Action:** “However, given multiple joint involvement, systemic comorbidities (including CVD, VTE, infections, postoperative joint dislocation, and osteoporosis) and polypharmacy, the benefits of these surgeries in terms of quality of life are not always clear among patients with RA ^12,15-17^.” **(Page 4, line 82-85, in the clean copy of the revised manuscript)**

References:

12	Burn, E. et al. The effect of rheumatoid arthritis on patient-reported outcomes following knee and hip replacement: evidence from routinely collected data. Rheumatology (Oxford) 58, 1016-1024, doi:10.1093/rheumatology/key409 (2019).

15	Momohara, S. et al. Efficacy of total joint arthroplasty in patients with established rheumatoid arthritis: improved longitudinal effects on disease activity but not on health-related quality of life. Mod Rheumatol 21, 476-481, doi:10.1007/s10165-011-0432-9 (2011).

16	Ravi, B. et al. A systematic review and meta-analysis comparing complications following total joint arthroplasty for rheumatoid arthritis versus for osteoarthritis. Arthritis Rheum 64, 3839-3849, doi:10.1002/art.37690 (2012).

17	Xiao, P. L. et al. Prevalence and treatment rate of osteoporosis in patients undergoing total knee and hip arthroplasty: a systematic review and meta-analysis. Arch Osteoporos 17, 16, doi:10.1007/s11657-021-01055-9 (2022).

**Comment 8** The authors did not mention the rate of infection in RA patients. Why not? It could be related or cause of mortality? Nothing was written.

**Response:** We thank the reviewer for highlighting the issue of postoperative infection risk in RA patients. We agree that surgical-site infections are important complications. However, our study was intentionally focused on long-term outcomes after arthroplasty (such as overall survival and cardiovascular events) rather than on early postoperative complications. In particular, our analysis tracked outcomes well beyond the first postoperative year, whereas most postoperative infections occur much earlier. For context, previous studies have shown that only about 27% of periprosthetic infections are diagnosed within 30 days of surgery and roughly 65% are identified within one year ^1^. In other words, the majority of infections are early events. Because our cohort analysis emphasized outcomes after this early period, we did not include acute infection rates as a primary endpoint.

It should also be noted that the infection risk in RA patients undergoing joint replacement has already been extensively studied in the literature. For example, Chung et al. (2021) examined over 1000 RA patients undergoing total knee arthroplasty and reported very similar 90-day and 1-year infection rates in RA versus osteoarthritis (OA) patients (2.58% vs. 2.66%) ^2^. In that study, the odds of acute infection were not significantly higher for RA (odds ratios around 0.8 and 0.46) compared to OA ^2^. Other analyses have found mixed results: some national cohort studies reported modestly higher infection rates in RA patients ^3-5^, while others saw no significant difference in early infection. In sum, multiple reports have already characterized infection rates and risk factors in RA arthroplasty patients (for instance, Lee et al. ^4^ and Ravi et al. ^6^), so we aimed to avoid duplicating that well-trodden ground.

Nevertheless, we fully appreciate the reviewer’s point that infection could influence overall outcomes. In the revised manuscript, we will explicitly acknowledge this limitation. We will add a sentence to the Discussion noting that postoperative infection – a predominantly short-term complication – was not assessed in our study, and we will cite representative references on this topic. By doing so, we will clarify that while infections are important, our study’s scope was on longer-term prognosis. Thank you again for this valuable suggestion, we will ensure that the absence of infection data is noted as a limitation and provide relevant citations in the revised paper.

References:

1	Pulido, L., Ghanem, E., Joshi, A., Purtill, J. J. & Parvizi, J. Periprosthetic joint infection: the incidence, timing, and predisposing factors. Clinical orthopaedics and related research 466, 1710-1715, doi:10.1007/s11999-008-0209-4 (2008).

2	Chung, H. K., Wen, S. H., Chang, W. C. & Liu, K. L. Acute surgical site infection after total knee arthroplasty in patients with rheumatoid arthritis versus osteoarthritis. Scientific reports 11, 22704, doi:10.1038/s41598-021-02153-x (2021).

3	Cordtz, R. L. et al. Predictors of revision, prosthetic joint infection and mortality following total hip or total knee arthroplasty in patients with rheumatoid arthritis: a nationwide cohort study using Danish healthcare registers. Annals of the rheumatic diseases 77, 281-288, doi:10.1136/annrheumdis-2017-212339 (2018).

4	Lee, D. K., Kim, H. J., Cho, I. Y. & Lee, D. H. Infection and revision rates following primary total knee arthroplasty in patients with rheumatoid arthritis versus osteoarthritis: a meta-analysis. Knee Surg Sports Traumatol Arthrosc 25, 3800-3807, doi:10.1007/s00167-016-4306-8 (2017).

5	Schrama, J. C. et al. Risk of revision for infection in primary total hip and knee arthroplasty in patients with rheumatoid arthritis compared with osteoarthritis: a prospective, population-based study on 108,786 hip and knee joint arthroplasties from the Norwegian Arthroplasty Register. Arthritis care & research 62, 473-479, doi:10.1002/acr.20036 (2010).

6	Ravi, B. et al. Increased risk of complications following total joint arthroplasty in patients with rheumatoid arthritis. Arthritis & rheumatology (Hoboken, N.J.) 66, 254-263, doi:10.1002/art.38231 (2014).

**Action:** “Fifth, we did not assess short-term postoperative complications (e.g., infections), future studies should be specifically designed to systematically investigate these outcomes.” **(Page 19, line 392-394, in the clean copy of the revised manuscript)**

**Comment 9** Row 94 and row 97: What it means “all cause mortality”? It is suggested to discriminate the main causes of death, included infection.

**Response:** All-cause mortality quantifies the total number of deaths in a population during a specified timeframe, irrespective of specific causes. It serves as an unbiased endpoint in epidemiological and clinical research by encompassing deaths from all origins, including diseases, accidents, and indirect health consequences. All-cause mortality is one of the most important indicators for the net risk-benefit effect of any clinical treatment regimens. This metric is prioritized over cause-specific mortality due to its methodological robustness. Cause-specific mortality relies on accurate attribution of death etiology, which is often compromised by diagnostic uncertainties, comorbidities, or multifactorial pathways (e.g., distinguishing cardiovascular deaths in individuals with diabetes). Such misclassification biases are circumvented when using all-cause mortality, ensuring a more reliable assessment of interventions or exposures. Furthermore, all-cause mortality captures the net impact of health interventions, including unintended adverse effects (e.g., a cancer drug reducing tumor-related deaths but increasing cardiovascular fatalities). It also enhances statistical power through higher event rates and inherently accounts for competing risks, such as deaths from unrelated causes in populations with chronic conditions. By reflecting the aggregate burden of mortality, it aligns with public health priorities to evaluate holistic survival outcomes rather than isolated disease-specific effects, making it a gold-standard metric for policy and comparative effectiveness research. Furthermore, due to limitations in the available data within the database, further analysis of the disease-specific mortality (e.g., infection-related mortality) was not feasible. We have provided a clarification in the **Discussion** section of the manuscript to acknowledge this limitation. We hope this explanation addresses your concern and clarifies our methodology.

**Action:** “Second, lack of sufficient information on the precise cause of death from some subjects precluded us from investigating the association of knee or hip arthroplasty with the risk of the cause-specific death. Nevertheless, the overall all-cause mortality trends are critically important in their own right, as mortality represents the overall net health outcome of various benefits and risks associated with disease management ^18^.” **(Page 18-19, line 380-385, in the clean copy of the revised manuscript)**

References:

18	Prasad, V. But how many people died? Health outcomes in perspective. Cleve Clin J Med 82, 146-150, doi:10.3949/ccjm.82a.14058 (2015).

**Comment 10** Which classification system was used to determine the level of RA? Nothing was written.

**Response:** We appreciate the reviewer’s suggestion. The IMRD database we utilized does not include the level of RA. Therefore, we regret that an analysis and report on the level of RA cannot be conducted. We have added a clarification in the **Discussion** section of the manuscript to acknowledge this limitation. We appreciate your understanding of the constraints inherent to the database and hope this explanation clarifies our approach.

**Action:** “First, population-based studies such as those performed in IMRD often lack information on disease activity measures and disease characteristics. Although our study was restricted among subjects with RA, we were unable to assess the stage or gravity of disease between patients with and without knee or hip arthroplasty due to the constraints of the database used in this study. It is possible that patients with more severe disease have increased all-cause mortality and risk of CVD.” **(Page 18, line 369-375, in the clean copy of the revised manuscript)**

**Comment 11** Which were the “potential confounders” factors?

**Response:** We appreciate the reviewer’s comment. The potential confounding factors primarily encompass RA duration, sociodemographic factors (including age at time of knee or hip arthroplasty, sex, BMI, and socioeconomic status as measured by the Townsend Deprivation Index), lifestyle factors (smoking status and alcohol consumption), and comorbidities (specifically the Charlson Comorbidity Index and individual conditions such as angina, atrial fibrillation, congestive heart failure, hypertension, ischaemic heart disease, stroke, transient ischaemic attack, myocardial infarction, valvular heart disease, venous thromboembolism, varicose veins, peripheral vascular disease, other circulatory disorders, dementia, depression, seizure disorders, chronic obstructive pulmonary disease, pneumonia or other infections, diabetes mellitus, hyperlipidaemia, osteoporosis, gastritis, gastroesophageal reflux disease, peptic ulcer disease, hepatic disorders, chronic kidney disease, other inflammatory conditions, fall risk, hip fracture, traumatic injuries, and malignancies). While these variables were comprehensively operationalized and described in the **Methods** section, illustrative examples were selectively presented in the Introduction to enhance conceptual clarity and mitigate potential ambiguities for readers unfamiliar with epidemiological methodologies.

**Action:** “To address these knowledge gaps, we conducted propensity-score matched cohort studies among patients with RA to investigate the relationship of knee or hip arthroplasty with risks of all-cause mortality, CVD and VTE, respectively, while controlling for potential confounders (including age, sex, BMI, lifestyle factors, and other comorbidities).” **(Page 5-6, line 106-110, in the clean copy of the revised manuscript)**

**Comment 12**

Methods

Row 122 -Why the authors included a broad spectrum for age? The results for TKA and THA between young and older patients are similar? I suggested to explain in more details.

**Response:** We appreciate the reviewer’s suggestion. We performed subgroup analyses stratified by 20-69 years and ≥ 70 years, with results demonstrating consistency with the overall findings. Previous investigations have documented that the mean age for arthroplasty procedures is approximately 70 years ^1-2^, and the risk of postoperative complications following knee arthroplasty shows a marked elevation commencing at age 70 years ^3^. This evidence collectively establishes 70 years as a clinically substantiated threshold for arthroplasty intervention. Consequently, our analytical framework adopted 70 years as the stratification criterion rather than employing the conventional dichotomization into younger and older adult cohorts.

References:

1	Riemer, K. & Lange, J. Early periprosthetic hip joint infection managed by cementless one-stage revision - a case series. J Bone Jt Infect 7, 43-50, doi:10.5194/jbji-7-43-2022 (2022).

2	Schnetzke, M., Wittmann, T., Raiss, P. & Walch, G. Short-term results of a second generation anatomic short-stem shoulder prosthesis in primary osteoarthritis. Arch Orthop Trauma Surg 139, 149-154, doi:10.1007/s00402-018-3039-1 (2019).

3	Easterlin, M. C., Chang, D. G., Talamini, M. & Chang, D. C. Older age increases short-term surgical complications after primary knee arthroplasty. Clinical orthopaedics and related research 471, 2611-2620, doi:10.1007/s11999-013-2985-8 (2013).

**Comment 13** Row 123-124: The RA codes was the same in the period of study (1995-2018)? Nothing was written.

**Response:** Many thanks for the reviewer’s suggestions. The data we analysed were sourced from IQVIA Medical Research Data (IMRD). The Read classification system is used to code specific diagnoses ^1^. Previous study has demonstrated the validity of the IMRD database in clinical and epidemiological research ^2^. Read codes for RA have been previously verified in the UK General Practice Research Database, with a positive predictive value (PPV) of around 80%. Approximately 60% of subjects in the UK General Practice Research Database overlap with IMRD ^3,4^. Study participants were identified through Read code, with RA diagnostic codes maintaining coding consistency throughout the entire study period (1995-2018). This methodological approach ensured standardized case ascertainment across the longitudinal observation window.

References:

1	Chisholm, J. The Read clinical classification. BMJ 300, 1092, doi:10.1136/bmj.300.6732.1092 (1990).

2	Lewis, J. D., Schinnar, R., Bilker, W. B., Wang, X. & Strom, B. L. Validation studies of the health improvement network (THIN) database for pharmacoepidemiology research. Pharmacoepidemiol Drug Saf 16, 393-401, doi:10.1002/pds.1335 (2007).

3	Rodriguez, L. A., Tolosa, L. B., Ruigomez, A., Johansson, S. & Wallander, M. A. Rheumatoid arthritis in UK primary care: incidence and prior morbidity. Scand J Rheumatol 38, 173-177, doi:10.1080/03009740802448825 (2009).

4	Watson, D. J., Rhodes, T., Cai, B. & Guess, H. A. Lower risk of thromboembolic cardiovascular events with naproxen among patients with rheumatoid arthritis. Arch Intern Med 162, 1105-1110, doi:10.1001/archinte.162.10.1105 (2002).

**Comment 14** Row 140,141 and 143: What it means the term “other”? I suggest to describe with more information.

**Response:** We appreciate the reviewer’s constructive feedback. The term “other circulatory diseases” refers to a category encompassing over 900 distinct circulatory conditions, including but not limited to acute rheumatic pericarditis, acute rheumatic endocarditis, chronic rheumatic mediastinopericarditis, and kyphoscoliotic heart disease. Similarly, “other Infections” refers to a classification of more than 1,000 infectious diseases, such as pneumocystis jirovecii pneumonia, kaposi sarcoma herpesvirus -associated syndromes, and related entities. The term “other inflammatory conditions” aggregates over 1,000 inflammatory disorders, including KSHV-inflammatory cytokine syndrome, gallbladder cancer -related inflammation, and fungal neglected tropical diseases. Due to the limited number of cases identified through Read codes for these specific conditions, we consolidated them into broader categories (other circulatory diseases, other Infections, and other inflammatory conditions) to enhance analytical validity and mitigate statistical biases arising from sparse data.

**Comment 15** Row 155-158: How the authors correlated the causality among death with joint arthroplasty in RA patients? It remains unclear.

**Response:** We sincerely appreciate the reviewer’s valuable suggestion. While our primary focus has been on examining the relationship of arthroplasty to all-cause mortality among patients with RA, we recognize that the limitations of the IMRD database, particularly regarding cause-of-death specificity, precluded more granular analysis of disease-specific mortality (e.g., mortality directly attributable to arthroplasty). We have provided a clarification in the **Discussion** section of the manuscript to acknowledge this limitation. We hope this clarification adequately addresses your thoughtful concern while maintaining the scientific integrity of our current analytical approach. Your insightful feedback will inform our future research directions in this critical area of investigation.

**Comment 16** The use and tourniquet, surgical time for TKA, type of implants (constrained or not), cementation or uncementation, use of elastic stocking, VTE drug prophylaxis were not mentioned in this section. Why not?

**Response:** We are thankful for the reviewer’s input. IMRD is an electronic medical record database of records of general practitioners (GPs) in the UK and represents the UK population in demographics and medical conditions. IMRD contains anonymized medical records from 790 general practices with approximately 17 million patients. The computerized information includes socio-demographics, anthropometrics, lifestyle factors, details from visits to GPs, diagnoses from specialists’ referrals and hospital admissions. And this type of database does not provide detailed records of specific surgical information. The National Institute for Health and Care Excellence (NICE) in the UK (https://www.nice.org.uk/), British Orthopaedic Association (https://www.boa.ac.uk/) and Royal College of Surgeons (https://www.rcseng.ac.uk/) have released a series of guidelines regarding joint replacement surgeries. The National Joint Registry (https://www.njrcentre.org.uk/) has also collected data related to joint replacement surgeries to provide feedback on surgical outcomes and quality. The standards for these types of surgeries are relatively uniform, which helps to reduce the bias caused by these factors on the results of our study. We have added a clarification in the **Discussion** section of the manuscript to acknowledge this limitation.

**Action:** “Furthermore, due to the limitations of the IMRD database, we were unable to include data on the clinical pathways before arthroplasty, VTE prophylaxis, preoperative cardiac evaluation, surgical details (e.g., tourniquet time, surgical time, type of implants, cementation or non-cementation and use of elastic stocking), and postoperative complications such as anemia and blood transfusions, which limits the scope of our analysis.” **(Page 18, line 375-380, in the clean copy of the revised manuscript)**

**Comment 17** The grade and stage of disease related with RA patients, were not written. It is suggested to add more information.

**Response:** Many thanks for the reviewer’s suggestion. As described above, IMRD is a suboptimal data source for studying grade and stage of RA since it lacks sufficiently detailed lab data to make inferences about grade and stage of disease. Consequently, we could not undertake a comprehensive analysis and report on the grade and stage of RA. We have provided a clarification in the **Discussion** section of the manuscript to acknowledge this limitation.

**Action:** “First, population-based studies such as those performed in IMRD often lack information on disease activity measures and disease characteristics. Although our study was restricted among subjects with RA, we were unable to assess the stage or gravity of disease between patients with and without knee or hip arthroplasty due to the constraints of the database used in this study. It is possible that patients with more severe disease have increased all-cause mortality and risk of CVD.” **(Page 18, line 369-375, in the clean copy of the revised manuscript)**

**Comment 18**

Results

The postoperative time after TKA and THA related with primary and secondary outcome were not written in the descriptive manner. Why not?

**Response:** We appreciate the reviewer’s suggestion. The postoperative periods following TKA or THA related with primary or secondary outcomes have been comprehensively documented in the **Table 1**-**Table 3**. To preclude potential misinterpretation by readers, a detailed elaboration of these postoperative periods has been explicitly addressed within the main body of the text.

**Action:** “The mean follow-up period was 5.9 years for the knee arthroplasty group and 5.5 years for the non-knee arthroplasty group. Participants with knee arthroplasty had a 23% lower risk of all-cause mortality compared with those without knee arthroplasty (HR=0.77, 95% CI 0.65 to 0.90; ARR=1.71%) (**Table 1**; **Fig 1A**). We identified 366 deaths (40.6 per 1,000 person-years) in subjects with hip arthroplasty and 395 deaths in those without hip arthroplasty (43.9 per 1,000 person-years) during follow-up. The mean follow-up period was 5.4 years for the hip arthroplasty group and 5.4 years for the non-hip arthroplasty group.” **(Page 10, line 217-225, in the clean copy of the revised manuscript)**

“The mean follow-up period was 5.7 years for the knee arthroplasty group and 5.3 years for the non-knee arthroplasty group. Participants with knee arthroplasty had a 14% lower, albeit non-statistically significant, risk of CVD than their counterparts (HR=0.86, 95% CI 0.73 to 1.01; ARR=0.09%) (**Table 2**; **Fig 2A**). During the follow-up period, 135 cases of CVD in the hip arthroplasty group and 146 CVD cases in the comparison group were identified. Incidence rates per 1,000 person-years for the hip arthroplasty and comparison cohorts were 17.6 and 19.6, respectively. The mean follow-up period was 5.2 years for the hip arthroplasty group and 5.1 years for the non-hip arthroplasty group.” **(Page 12, line 248-256, in the clean copy of the revised manuscript)**

“The mean follow-up period was 5.8 years for the knee arthroplasty group and 5.6 years for the non-knee arthroplasty group. Compared with those without knee arthroplasty, the HR of VTE for subjects with knee arthroplasty was 1.63 (95% CI 1.23 to 2.17) and the corresponding ARR was 0.97% (**Table 3**; **Fig 3A**). Similar results were also observed when the association between hip arthroplasty and the risk of VTE was examined (7.98 vs. 3.88 per 1,000 person-years). The mean follow-up period was 5.3 years for the hip arthroplasty group and 5.2 years for the non-hip arthroplasty group.” **(Page 14, line 277-284, in the clean copy of the revised manuscript)**

**Comment 19** The correlation between BMI and VTE were not mentioned? It is suggested to add more information.

**Response:** Many thanks for the reviewer’s suggestions. The primary objective of this study was to examine the relationship of knee arthroplasty or hip arthroplasty to all-cause mortality, risk of CVD and incident VTE among patients with RA. In our analytical approach, BMI was systematically adjusted as a confounding variable in all statistical models to account for its potential mediating effects. This investigation did not extend to examining the direct relationship between BMI and VTE. This methodological clarification is provided to ensure precise understanding of our study’s scope and analytical boundaries, particularly regarding variable selection and adjustment strategies. We anticipate that this elucidation satisfactorily resolves the raised inquiry while enhancing the interpretability of our research methodology.

**Comment 20** The use of medications*like corticoids) and period of time related with RA were not mentioned? It is suggested to explain in more details.

**Response:** We extend our gratitude to the reviewer for their constructive commentary. The use of medications has been comprehensively documented in the baseline characteristics table included within the supplementary materials. IMRD often lack comprehensive information on therapeutic parameters such as period of biological disease-modifying anti-rheumatic drugs administration. We have added a clarification in the **Discussion** section of the manuscript to acknowledge this limitation.

**Action:** “Third, the current data set does not contain information on the period of biological disease-modifying anti-rheumatic drugs (DMARDs) administration. Since studies have shown that biological DMARDs use was associated a decreased risk of mortality and cardiovascular events in patients with RA ^64,65^, without adjustment of biological DMARD could also introduce potential residual confounding.” **(Page 19, line 385-391, in the clean copy of the revised manuscript)**

References:

64	Rodriguez-Rodriguez, L. et al. Treatment in rheumatoid arthritis and mortality risk in clinical practice: the role of biologic agents. Clin Exp Rheumatol 34, 1026-1032 (2016).

65	Greenberg, J. D. et al. Tumour necrosis factor antagonist use and associated risk reduction of cardiovascular events among patients with rheumatoid arthritis. Ann Rheum Dis 70, 576-582 (2011).

**Comment 21** The clinical pathways before THA and TKA between 1995 and 2018 were not written. Why not?

**Response:** We sincerely appreciate the reviewer’s valuable feedback. Regrettably, IMRD does not contain the clinical pathways before THA and TKA between 1995 and 2018, and therefore cannot provide the requested information. In response to this limitation, we have incorporated a clarification within the **Discussion** section of our manuscript to explicitly acknowledge this data constraint. We hope this clarification has been helpful in addressing your concerns and providing insight into how we developed our methodology.

**Action:** “Furthermore, due to the limitations of the IMRD database, we were unable to include data on the clinical pathways before arthroplasty, VTE prophylaxis, preoperative cardiac evaluation, surgical details (e.g., tourniquet time, surgical time, type of implants, cementation or non-cementation and use of elastic stocking), and postoperative complications such as anemia and blood transfusions, which limits the scope of our analysis.” **(Page 18, line 375-380, in the clean copy of the revised manuscript)**

**Comment 22**

Tables 1,2,3

Why the authors separated the variable age above and under 70ys? It is suggested to add references to support.

**Response:** We appreciate the reviewer’s suggestion. Previous studies reported that the average age for undergoing arthroplasty was 70 years ^1,2^ and the risk of experiencing any complication from knee arthroplasty begins to increase at age 70 years ^3^. 70 years old is a relatively reasonable arthroplasty cut-off. Therefore, we choose 70 years old for the analysis. We hope this response has offered helpful perspectives in addressing your inquiries and providing insight into how we developed our methodology.

References:

1	Riemer, K. & Lange, J. Early periprosthetic hip joint infection managed by cementless one-stage revision - a case series. J Bone Jt Infect 7, 43-50, doi:10.5194/jbji-7-43-2022 (2022).

2	Schnetzke, M., Wittmann, T., Raiss, P. & Walch, G. Short-term results of a second generation anatomic short-stem shoulder prosthesis in primary osteoarthritis. Arch Orthop Trauma Surg 139, 149-154, doi:10.1007/s00402-018-3039-1 (2019).

3	Easterlin, M. C., Chang, D. G., Talamini, M. & Chang, D. C. Older age increases short-term surgical complications after primary knee arthroplasty. Clinical orthopaedics and related research 471, 2611-2620, doi:10.1007/s11999-013-2985-8 (2013).

**Comment 23**

Discussion

This section is short. It can be improved

**Response:** We would like to express our sincere gratitude for the reviewer’s insightful comments and constructive suggestions. In response to these valuable inputs, we have carefully revised the manuscript to expand the discussion section substantially, aiming to enhance the depth and clarity of our interpretation of the research findings.

**Action:** We have revised the **Discussion** section. **(Page 15-22, line 301-475, in the clean copy of the revised manuscript)**

**Comment 24** the main findings were written in the first paragraph in the generic manner. It can be improved.

**Response:** We appreciate the reviewer’s suggestion. We have modified the the **Discussion** according to your suggestion.

**Action:** “In this general practice cohort representative of the UK population, pioneering the exploration of knee or hip arthroplasty’s impact on all-cause mortality, CVD risk, and incident VTE in patients with RA, we identified significant associations between joint replacement surgery and critical health outcomes. Our findings showed a substantially lower risk of all-cause mortality (23% for KA, 13% for HA) in patients with RA after knee or hip arthroplasty, indicating significant protective associations for both KA and HA regarding all-cause mortality. Our study revealed that both KA and HA demonstrated cardioprotective properties in patients with RA, exhibiting a trend toward reduced cardiovascular disease risk (14% for KA, 16% for HA). However, this potential cardiovascular benefit must be weighed against a substantial elevation in VTE risk, with postoperative analyses showing 63% and 119% increased incidence rates for KA and HA recipients respectively. These results were consistent across age and sex strata. Our findings address a critical gap in the surgical outcomes literature for RA populations and carry important clinical implications for risk-benefit assessments in perioperative decision-making.” **(Page 15-16, line 302-316, in the clean copy of the revised manuscript)**

**Comment 25** The subgoup analysis and correlation with risk factors could be more explored.

**Response:** Many thanks for the reviewer’s suggestion. Per the reviewer’s comment, we have added the corresponding content to the **Discussion** section.

**Action:** “Our subgroup analyses revealed that the associations of knee arthroplasty or hip arthroplasty with the risk of mortality, CVD, and VTE were consistent across the age and sex strata. Specifically, no significant heterogeneity was observed between patients aged < 70 years and those ≥ 70 years, nor between male and female subgroups, aligning with the overall cohort trends. These findings suggest that RA-specific systemic factors, rather than age- or sex-related biological differences, predominantly drive postoperative complications in this population. The uniformity in risk trends across age subgroups may reflect the persistent systemic inflammatory burden and immune dysregulation inherent to RA pathophysiology, which exacerbates postoperative thrombogenic and atherosclerotic pathways. Chronic elevation of inflammatory markers such as C-reactive protein (CRP) and interleukin-6 (IL-6), commonly observed in RA, promotes endothelial dysfunction and hypercoagulability, thereby amplifying CVD and VTE risks irrespective of age ^66^. Similarly, the lack of sex-based differences aligns with evidence that RA-related immune-mediated mechanisms (e.g., synovial pannus formation, cytokine-driven osteoclast activation) override sex-specific hormonal or metabolic modifiers in shaping postoperative outcomes ^67,68^. Perioperative factors further explain the consistent trends. Patients with RA undergoing joint replacement often exhibit prolonged immobilization, heightened surgical trauma, and frequent blood transfusions—universal risk enhancers for VTE and cardiovascular stress ^69^. Additionally, immunosuppressive therapies (e.g., methotrexate, biologics) used pre- and postoperatively may uniformly modulate infection and thrombosis risks across demographics ^70,71^. These results underscore the critical balance required in clinical practice between pursuing long-term prognostic advantages through surgical intervention and mitigating acute perioperative risks. Such considerations should be thoroughly integrated into shared decision-making processes to optimize patient outcomes.” **(Page 20-21, line 412-438, in the clean copy of the revised manuscript)**

References:

66	de Araujo, L. C. T., Westerholt, A., Sandiford, A. N., Gursche, A. & Kendoff, D. Periprosthetic joint infections in patients with rheumatoid arthritis are associated with higher complication and mortality rates. Arch Orthop Trauma Surg 144, 5101-5109, doi:10.1007/s00402-024-05248-y (2024).

67	Alivernini, S. et al. Distinct synovial tissue macrophage subsets regulate inflammation and remission in rheumatoid arthritis. Nature medicine 26, 1295-1306, doi:10.1038/s41591-020-0939-8 (2020).

68	McHugh, K. P. et al. The role of cell-substrate interaction in regulating osteoclast activation: potential implications in targeting bone loss in rheumatoid arthritis. Annals of the rheumatic diseases 69 Suppl 1, i83-85, doi:10.1136/ard.2009.120188 (2010).

69	Akkara Veetil, B. M. & Bongartz, T. Perioperative care for patients with rheumatic diseases. Nat Rev Rheumatol 8, 32-41, doi:10.1038/nrrheum.2011.171 (2011).

70	Bartoloni, E. et al. Immunomodulation for accelerated atherosclerosis in rheumatoid arthritis and systemic lupus erythematosus. Autoimmunity reviews 24, 103760, doi:10.1016/j.autrev.2025.103760 (2025).

71	Yeganeh, M. H., Kheir, M. M., Shahi, A. & Parvizi, J. Rheumatoid Arthritis, Disease Modifying Agents, and Periprosthetic Joint Infection: What Does a Joint Surgeon Need to Know? The Journal of arthroplasty 33, 1258-1264, doi:10.1016/j.arth.2017.11.031 (2018).

**Comment 26** The VTE prophylaxis included mechanical and medications were not written in this section. Why not?

**Response:** We appreciate the reviewer’s suggestion. Per the reviewer’s comment, we have added the corresponding content to the **Discussion** section.

**Action:** “Meanwhile, VTE prophylaxis in patients with RA undergoing arthroplasty integrates mechanical and pharmacological approaches ^79^. Mechanical strategies, including intermittent pneumatic compression and graduated compression stockings, reduce venous stasis and are preferred for high-bleeding-risk patients, while anticoagulants such as low-molecular-weight heparins and direct oral anticoagulants demonstrate efficacy in lowering VTE incidence with favorable safety profiles ^80,81^. Multimodal prophylaxis, guided by risk assessment tools like Caprini or RAPT, optimizes outcomes by balancing thromboprophylaxis benefits against bleeding risks, particularly in high-risk RA cohorts with chronic inflammation or immobilization ^82^. Tailoring regimens to individual factors (e.g., renal function, drug interactions) remains critical to ensuring safety and adherence ^83^.” **(Page 22, line 461-472, in the clean copy of the revised manuscript)**

References:

79	Bartlett, M. A., Mauck, K. F., Stephenson, C. R., Ganesh, R. & Daniels, P. R. Perioperative Venous Thromboembolism Prophylaxis. Mayo Clin Proc 95, 2775-2798, doi:10.1016/j.mayocp.2020.06.015 (2020).

80	Afshari, A., Fenger-Eriksen, C., Monreal, M., Verhamme, P. & Force, E. V. G. T. European guidelines on perioperative venous thromboembolism prophylaxis: Mechanical prophylaxis. Eur J Anaesthesiol 35, 112-115, doi:10.1097/EJA.0000000000000726 (2018).

81	Frostick, S. Pharmacological thromboprophylaxis and total hip or knee replacement. Br J Nurs 25, 45-53, doi:10.12968/bjon.2016.25.1.45 (2016).

82	Schunemann, H. J. et al. American Society of Hematology 2018 guidelines for management of venous thromboembolism: prophylaxis for hospitalized and nonhospitalized medical patients. Blood Adv 2, 3198-3225, doi:10.1182/bloodadvances.2018022954 (2018).

83	Flevas, D. A. et al. Thromboembolism prophylaxis in orthopaedics: an update. EFORT Open Rev 3, 136-148, doi:10.1302/2058-5241.3.170018 (2018).

**Comment 27** The period of hospitalization and correlation with primary and secondary outcomes remains undetermined,

**Response:** We appreciate the reviewer’s suggestion. Per the reviewer’s comment, we have added the corresponding content to the **Discussion** section.

**Action:** “In patients with RA undergoing joint arthroplasty, prolonged hospitalization due to factors such as extended immobilization, systemic inflammatory burden, comorbidities, rehabilitation demands, and surgical stress may elevate risks of mortality, CVD, and VTE ^76,77^. This necessitates prompt implementation of thromboprophylaxis, cardiovascular risk mitigation strategies, and early mobilization protocols. Perioperative management must be rigorously optimized to reduce complications associated with extended hospital stays in this clinically vulnerable population ^77,78^.” **(Page 21-22, line 454-461, in the clean copy of the revised manuscript)**

References:

76	Nikiphorou, E. et al. The Effect of Disease Severity and Comorbidity on Length of Stay for Orthopedic Surgery in Rheumatoid Arthritis: Results from 2 UK Inception Cohorts, 1986-2012. J Rheumatol 42, 778-785, doi:10.3899/jrheum.141049 (2015).

77	Morse, K. W. et al. Factors Associated With Increasing Length of Stay for Rheumatoid Arthritis Patients Undergoing Total Hip Arthroplasty and Total Knee Arthroplasty. HSS J 18, 196-204, doi:10.1177/15563316221076603 (2022).

78	Nguyen-Oghalai, T. U. et al. The impact of rheumatoid arthritis on rehabilitation outcomes after lower extremity arthroplasty. J Clin Rheumatol 13, 247-250, doi:10.1097/RHU.0b013e3181570ad4 (2007).

**Comment 28** The clinical relevance could be emphasized.

**Response:** We are thankful for the reviewer’s feedback and have revised the corresponding content in the **Discussion** section to emphasize the clinical relevance.

**Action:** “This is the first study which showed knee and hip arthroplasty was associated with a lower risk of mortality. It has important implications for facilitating clinical decision making, suggesting that timely surgical intervention may confer systemic health benefits beyond joint function restoration. In addition, our findings further suggested that both knee and hip arthroplasty have cardioprotective benefits for patients with RA. These findings provide further justification for increased attention to the impact of treatments directed towards RA-related disability in the prevention and management of other common chronic conditions including CVD. Moreover, identification of a higher risk of VTE after knee or hip arthroplasty among patients with RA will help with appropriate perioperative planning and post-operative follow-up and monitoring.” **(Page 19-20, line 401-412, in the clean copy of the revised manuscript)**

“These results underscore the critical balance required in clinical practice between pursuing long-term prognostic advantages through surgical intervention and mitigating acute perioperative risks. Such considerations should be thoroughly integrated into shared decision-making processes to optimize patient outcomes.” **(Page 21, line 434-438, in the clean copy of the revised manuscript)**
