# Supplementary material for: Reduced mortality but elevated venous thromboembolism risk following knee and hip arthroplasty in patients with rheumatoid arthritis: A general population-based cohort study
Source: PLoS One. 2025 Nov 7;20(11):e0335453. doi: 10.1371/journal.pone.0335453 (PMC12594356; doi:10.1371/journal.pone.0335453)
Supplement: Attachment — Submitted filename: Response_to_Reviewers_auresp_2.docx [file pone.0335453.s005.docx]

**Editors’ Comments to the Authors:**

**Comment 1** After careful consideration, we feel that it has merit but does not fully meet PLOS ONE’s publication criteria as it currently stands. Therefore, we invite you to submit a revised version of the manuscript that addresses the points raised during the review process.

**Response:** We appreciate the editor’s comment. We provide a point-by-point response to the reviewers’ comments. We hope that our responses are satisfactory and that the changes we have made in the text reflect our responsiveness to the comments and suggestions.

**Comment 2** Several reviewers (notably Reviewers 2 and 5) have made detailed and constructive suggestions regarding the structure and content of the Abstract, Introduction, and Methods. Reviewer 2 emphasized the need to include additional epidemiological data (prevalence and mortality related to cardiovascular disease and venous thromboembolism in RA patients undergoing arthroplasty) and to clarify how cardiovascular and VTE diagnoses were determined. Reviewer 5 raised similar points about clarifying definitions, coding, and the inclusion criteria, and also stressed the need to adjust the title to reflect only statistically supported findings. These recommendations are consistent and should be addressed in the revision.

**Response:** Many thanks for the editor’s and reviewers’ suggestion. In light of the valuable feedback from the reviewers, we have made revisions to the structure and content of the **Abstract**, **Introduction**, and **Methods** sections. We have included requested epidemiological data, clarified the definitions and methodologies for cardiovascular/VTE diagnoses and inclusion criteria, and adjusted the title to reflect only statistically supported findings.

**Comment 3** Reviewer 4 raised specific concerns about data presentation, including inconsistencies in patient numbers between tables and unclear statements about procedures performed. These issues are important for the transparency and credibility of the results, and I recommend you address them directly.

**Response:** We sincerely thank Reviewer 4 for the insightful comments. We have conducted a thorough re-examination of the dataset and the corresponding descriptions in the manuscript. All patient numbers have been carefully cross-checked and harmonized between the text and the tables to ensure complete consistency. Furthermore, the descriptions of the **Methods** and **Results** have been revised to be more precise and unambiguous. We believe these revisions have significantly enhanced the transparency and accuracy of our data presentation, and we have detailed all changes within the revised manuscript.

**Comment 4** In some cases, reviewer suggestions diverge. For example, Reviewer 2 requested substantial additional intraoperative and subgroup analyses (tourniquet time, medications, subgrouping by BMI, gender, OA grade), whereas Reviewer 5 focused more on clarifying methodology, inclusion criteria, and the interpretation of results without requiring extensive new data collection. Given the nature of your study, which is based on a pre-existing database, some of Reviewer 2’s requests for additional intraoperative variables may not be feasible. In this case, I recommend you clearly explain the limitations of the database and why certain variables could not be included, rather than attempting unrealistic additional analyses.

**Response:** We sincerely appreciate the editorial guidance on reconciling the reviewers' comments. In revising the manuscript, we will carefully address the points raised by Reviewer 5 regarding methodological clarification, inclusion criteria, and interpretation of results, as these align well with the scope and data availability of our study. For the more extensive analytical requests from Reviewer 2—such as additional intraoperative variables and subgroup analyses—we will transparently acknowledge the limitations of our pre-existing dataset, clarifying where certain variables (e.g., tourniquet time, specific medication data) were not systematically collected and are therefore unavailable for analysis. This approach allows us to strengthen the paper's validity while maintaining methodological realism, and we will incorporate these explanations into the **Discussion** section of the manuscript.

**Comment 5**

Overall, please focus your revision on:

Clarifying the title and aligning it with statistically supported results.

**Response:** We appreciate the editor’s comment. We have clarified the title and aligned it with statistically supported results.

**Action:** “Reduced mortality but elevated venous thromboembolism risk following knee and hip arthroplasty in patients with rheumatoid arthritis: a general population-based cohort study.” **(Page 1, line 1-3, in the clean copy of the revised manuscript)**

**Comment 6** Revising the Abstract for consistency and specificity, including appropriate key words.

**Response:** Many thanks for the editor’s comment. The **Abstract** has been revisied for consistency and specificity, including appropriate key words.

**Action:** “Arthroplasty is indicated for patients with rheumatoid arthritis (RA) who experience significant joint damage, including bone erosions, cartilage degradation and joint deformities. However, studies on its associations with all-cause mortality, cardiovascular disease (CVD), and venous thromboembolism (VTE) among patients with RA are scarce.” **(Page 2, line 27-31, in the clean copy of the revised manuscript)**

“**Key words**: Rheumatoid arthritis; Hip arthroplasty; Knee arthroplasty; Mortality; Cardiovascular disease; Venous thromboembolism” **(Page 3, line 62-63, in the clean copy of the revised manuscript)**

**Comment 7** Expanding the Introduction to address epidemiology and the rationale for the study.

**Response:** Many thanks for the editor’s comment. We have expanded the **Introduction** to address epidemiology and the rationale for the study.

**Action:** “It is associated with increased risks of cardiovascular disease (CVD, 48%, pooled risk ratio=1.48 (95% CI 1.36 to 1.62)) ^2,3^, venous thromboembolism (VTE, 100%, adjusted hazard ratio=2.0 (95% CI, 1.9-2.2)) ^4,5^, and mortality (43%, HR=1.43 (95% CI 1.28 to 1.59)) ^6,7^. Epidemiological data indicate that approximately 9% patients with RA are affected by CVD, corresponding with an incidence rate of 3.30 per 100 patient-years (95% CI 2.08–4.25) ^8^, while VTE occurs around 7.2% of these patients ^9^.” **(Page 4, line 67-73, in the clean copy of the revised manuscript)**

“Previous studies have reported that the rate of mortality after hip and knee arthroplasty surgery is up to 4.8%, with major causes including pulmonary embolism, myocardial infarction, stroke, surgical site infections, prosthetic joint infections, and complications related to anesthesia and bleeding ^19-22^.” **(Page 5, line 92-95, in the clean copy of the revised manuscript)**

“However, no study has compared VTE incidence in patients with RA undergoing joint arthroplasty with those without joint arthroplasty. The increased susceptibility to VTE in RA is thought to result from chronic systemic inflammation, endothelial dysfunction, reduced mobility, and the use of glucocorticoids or NSAIDs ^29-31^.” **(Page 5, line 107-111, in the clean copy of the revised manuscript)**

“All-cause mortality is one of the most important indicators for the net risk-benefit effect of any clinical treatment regimens ^18^. Previous studies have reported that the rate of mortality after hip and knee arthroplasty surgery is up to 4.8%, with major causes including pulmonary embolism, myocardial infarction, stroke, surgical site infections, prosthetic joint infections, and complications related to anesthesia and bleeding ^19-22^. However, for patients with RA, joint arthroplasty may significantly improve physical function, reduce pain, and enhance overall quality of life. By alleviating disability and decreasing long-term systemic inflammation, such interventions could potentially contribute to a reduction in all-cause mortality among this population over time ^23,24^. Therefore, the association of either knee or hip arthroplasty with all-cause mortality in patients with RA specifically remains understudied.” **(Page 5, line 90-101, in the clean copy of the revised manuscript)**

“Based on the anatomic location of their RA, patients with RA may undergo various surgical options, such as tenosynovectomy, radiosynovectomy, arthroscopic surgery, osteotomy, joint fusion, procedure for soft-tissue release, small-joint implant arthroplasty, metatarsal-head excision arthroplasty and total joint replacement ^14^.” **(Page 4, line 83-87, in the clean copy of the revised manuscript)**

“The aim of this investigation was to evaluate the relationship of knee arthroplasty or hip arthroplasty to all-cause mortality, risk of CVD and incident VTE among patients with RA. To address these knowledge gaps, we conducted propensity-score matched cohort studies among patients with RA to investigate the relationship of knee or hip arthroplasty with risks of all-cause mortality, CVD and VTE, respectively, while controlling for potential confounders (including age, sex, BMI, lifestyle factors, and other comorbidities). Additionally, we performed subgroup analyses (differential risks by age and sex) to explore potential residual confounding by indication. Based on these, we hypothesized that knee and hip arthroplasty may be associated with lower risks of mortality and CVD but an increased risk of VTE among patients with RA.” **(Page 6, line 117-127, in the clean copy of the revised manuscript)**

**Comment 8** Providing methodological clarity on case definitions, coding, inclusion/exclusion criteria, and database characteristics.

**Response:** We thank the editor for this insightful feedback. In response to the request for greater methodological clarity, we have provided methodological clarity on case definitions, coding, inclusion/exclusion criteria, and database characteristics. These amendments are incorporated to ensure the study's reproducibility and to bolster the transparency and rigor of our methodological approach.

**Action:** Revised the **Methods** section and the **Results** section. **(Page 6-16, line 129-320, in the clean copy of the revised manuscript)**

**Comment 9** Correcting data inconsistencies in tables and figures.

**Response:** Many thanks for the editor’s suggestions. The description in the **Results** section has been revised accordingly to mitigate potential ambiguities, specifically to clarify which patients underwent arthroplasty and which did not.

**Action:** Revised the **Results** section. **(Page 10-16, line 227-320, in the clean copy of the revised manuscript)**

**Comment 10** Shortening and refining the Discussion, ensuring that conclusions are balanced and do not imply causality where only associations have been demonstrated.

**Response:** We appreciate the valuable feedback. In revising this section, we have carefully shortened and refined the text to highlight the key findings and their context within the existing literature. Furthermore, we have ensured that our conclusions are appropriately balanced by consistently using language that reflects the observational nature of our study, clarifying that the results demonstrate associations rather than implying causal relationships. We believe these revisions will significantly strengthen the manuscript's clarity and scholarly rigor.

**Action:** Revised the **Discussion** section and the **Conclusion** section. **(Page 16-22, line 322-471, in the clean copy of the revised manuscript)**

**Comment 11** Explaining clearly in the manuscript any variables not available in the dataset and the implications for interpretation.

**Response:** We are thankful for the editor’s input. To uphold methodological rigor, we will transparently acknowledge the limitations of our dataset, particularly that the analysis could not incorporate certain variables due to their unsystematic record. This candor is crucial for interpreting the findings realistically and will be elaborated upon in the **Discussion** section to strengthen the study's overall validity.

**Action:** “Several limitations of this study are worth noting. First, population-based studies such as those performed in IMRD often lack information on disease activity measures and disease characteristics. Although our study was restricted among subjects with RA, we were unable to assess the stage or gravity of disease between patients with and without knee or hip arthroplasty due to the constraints of the database used in this study. It is possible that patients with more severe disease have increased all-cause mortality and risk of CVD. Furthermore, due to the limitations of the IMRD database, we were unable to include data on the clinical pathways before arthroplasty, radiographs for the assessment of joint injury severity, VTE prophylaxis, imaging for DVT, preoperative cardiac evaluation and anticoagulation controlled, surgical details (e.g., tourniquet time, surgical time, type of implants, cementation or non-cementation and use of elastic stocking), and postoperative complications such as anemia and blood transfusions, which limits the scope of our analysis. Second, lack of sufficient information on the precise cause of death from some subjects precluded us from investigating the association of knee or hip arthroplasty with the risk of the cause-specific death. Nevertheless, the overall all-cause mortality trends are critically important in their own right, as mortality represents the overall net health outcome of various benefits and risks associated with disease management 18. Third, the current data set does not contain information on the period of biological disease-modifying anti-rheumatic drugs (DMARDs) administration. Since studies have shown that biological DMARDs use was associated a decreased risk of mortality and cardiovascular events in patients with RA 69,70, without adjustment of biological DMARD could also introduce potential residual confounding. Fourthly, we are unable to distinguish between unilateral and bilateral cases, and therefore cannot further explore the distinct impacts of unilateral and bilateral procedures on the outcomes. Fifth, we did not assess short-term postoperative complications (e.g., infections), future studies should be specifically designed to systematically investigate these outcomes.” **(Page 18-19, line 384-411, in the clean copy of the revised manuscript)**

**Comment 12** Addressing these points will improve the clarity, transparency, and clinical relevance of your work. I look forward to receiving your revised manuscript.

**Response:** We sincerely thank the Editor for their constructive feedback and for the opportunity to revise our manuscript. We have carefully considered all the points raised and have diligently addressed each one in the revised version. The changes made aim to significantly enhance the clarity of our methodology, improve the transparency of our data presentation, and more strongly articulate the clinical relevance of our findings. We believe the manuscript has been substantially improved as a result of this process and we look forward to the Editor's assessment of the revised work.

**Reviewer #2:**

**Comment 1**

Abstract

Row 27-29: This phrase could be more specific according with the main goal.

**Response:** We thank the reviewer for this insightful comment. We have revised the opening sentence of the Abstract to more explicitly align with the primary objectives of our study. The phrase now specifically mentions the three key outcomes—all-cause mortality, cardiovascular disease, and venous thromboembolism—which are the focus of our investigation. This change better introduces the research gap our study aims to address.

**Action:** “Arthroplasty is indicated for patients with rheumatoid arthritis (RA) who experience significant joint damage, including bone erosions, cartilage degradation and joint deformities. However, studies on its associations with all-cause mortality, cardiovascular disease (CVD), and venous thromboembolism (VTE) among patients with RA are scarce.” **(Page 2, line 27-31, in the clean copy of the revised manuscript)**

**Comment 2** Key words: The authors could add more terms.

**Response:** Many thanks for the reviewer’s suggestion. We have added more terms in the **Key words** section.

**Action:** “**Key words**: Rheumatoid arthritis; Hip arthroplasty; Knee arthroplasty; Mortality; Cardiovascular disease; Venous thromboembolism” **(Page 3, line 62-63, in the clean copy of the revised manuscript)**

**Comment 3**

Introduction

It is lacking information (frequency and prevalence) about cardiovascular problems with RA and arthroplasty.

**Response:** We appreciate the reviewer’s comments and have added the corresponding content to the **Introduction** section.

**Action:** “It is associated with increased risks of cardiovascular disease (CVD, 48%, pooled risk ratio=1.48 (95% CI 1.36 to 1.62)) ^2,3^, venous thromboembolism (VTE, 100%, adjusted hazard ratio=2.0 (95% CI, 1.9-2.2)) ^4,5^, and mortality (43%, HR=1.43 (95% CI 1.28 to 1.59)) ^6,7^. Epidemiological data indicate that approximately 9% patients with RA are affected by CVD, corresponding with an incidence rate of 3.30 per 100 patient-years (95% CI 2.08–4.25) ^8^, while VTE occurs around 7.2% of these patients ^9^.” **(Page 4, line 67-73, in the clean copy of the revised manuscript)**

References:

2	Avina-Zubieta, J. A., Thomas, J., Sadatsafavi, M., Lehman, A. J. & Lacaille, D. Risk of incident cardiovascular events in patients with rheumatoid arthritis: a meta-analysis of observational studies. Annals of the rheumatic diseases 71, 1524-1529, doi:10.1136/annrheumdis-2011-200726 (2012).

3	Kitas, G. D. & Gabriel, S. E. Cardiovascular disease in rheumatoid arthritis: state of the art and future perspectives. Annals of the rheumatic diseases 70, 8-14, doi:10.1136/ard.2010.142133 (2011).

4	Holmqvist, M. E. et al. Risk of venous thromboembolism in patients with rheumatoid arthritis and association with disease duration and hospitalization. Jama 308, 1350-1356, doi:10.1001/2012.jama.11741 (2012).

5	Zoller, B., Li, X., Sundquist, J. & Sundquist, K. Risk of pulmonary embolism in patients with autoimmune disorders: a nationwide follow-up study from Sweden. Lancet 379, 244-249, doi:10.1016/S0140-6736(11)61306-8 (2012).

6	Holmqvist, M., Ljung, L. & Askling, J. Mortality following new-onset Rheumatoid Arthritis: has modern Rheumatology had an impact? Ann Rheum Dis 77, 85-91, doi:10.1136/annrheumdis-2017-212131 (2018).

7	Solomon, D. H. et al. Cardiovascular morbidity and mortality in women diagnosed with rheumatoid arthritis. Circulation 107, 1303-1307, doi:10.1161/01.cir.0000054612.26458.b2 (2003).

8	Peters, M. J. et al. Does rheumatoid arthritis equal diabetes mellitus as an independent risk factor for cardiovascular disease? A prospective study. Arthritis Rheum 61, 1571-1579, doi:10.1002/art.24836 (2009).

9	Liang, K. P. et al. Incidence of noncardiac vascular disease in rheumatoid arthritis and relationship to extraarticular disease manifestations. Arthritis Rheum 54, 642-648, doi:10.1002/art.21628 (2006).

**Comment 4** the main causes of mortality associated with arthroplasty were not written.

**Response:** We appreciate the reviewer’s comment. In accordance with the reviewer’s recommendation, we have added the corresponding content to the **Introduction** section.

**Action:** “Previous studies have reported that the rate of mortality after hip and knee arthroplasty surgery is up to 4.8%, with major causes including pulmonary embolism, myocardial infarction, stroke, surgical site infections, prosthetic joint infections, and complications related to anesthesia and bleeding ^19-22^.” **(Page 5, line 92-95, in the clean copy of the revised manuscript)**

References:

19	Ko, K. et al. Total Knee Arthroplasty: Is It Safe? A Single-Center Study of 4,124 Patients in South Korea. Clinics in orthopedic surgery 15, 935-941, doi:10.4055/cios22088 (2023).

20	Mantilla, C. B., Horlocker, T. T., Schroeder, D. R., Berry, D. J. & Brown, D. L. Frequency of myocardial infarction, pulmonary embolism, deep venous thrombosis, and death following primary hip or knee arthroplasty. Anesthesiology 96, 1140-1146, doi:10.1097/00000542-200205000-00017 (2002).

21	Parvizi, J. et al. Revision total hip arthroplasty in octogenarians. A case-control study. The Journal of bone and joint surgery. American volume 89, 2612-2618, doi:10.2106/JBJS.F.00881 (2007).

22	Pioli, G. et al. Predictors of mortality after hip fracture: results from 1-year follow-up. Aging Clin Exp Res 18, 381-387, doi:10.1007/BF03324834 (2006).

**Comment 5** The association of VTE and RA were poorly written in this section.

**Response:** We appreciate the reviewer’s suggestion. Following the reviewer’s advice, we have added the association of VTE and RA to the **Introduction** section.

**Action:** “It is associated with increased risks of cardiovascular disease (CVD, 48%, pooled risk ratio=1.48 (95% CI 1.36 to 1.62)) ^2,3^, venous thromboembolism (VTE, 100%, adjusted hazard ratio=2.0 (95% CI, 1.9-2.2)) ^4,5^, and mortality (43%, HR=1.43 (95% CI 1.28 to 1.59)) ^6,7^. Epidemiological data indicate that approximately 9% patients with RA are affected by CVD, corresponding with an incidence rate of 3.30 per 100 patient-years (95% CI 2.08–4.25) ^8^, while VTE occurs around 7.2% of these patients ^9^.” **(Page 4, line 67-73, in the clean copy of the revised manuscript)**

“However, no study has compared VTE incidence in patients with RA undergoing joint arthroplasty with those without joint arthroplasty. The increased susceptibility to VTE in RA is thought to result from chronic systemic inflammation, endothelial dysfunction, reduced mobility, and the use of glucocorticoids or NSAIDs ^29-31^.” **(Page 5, line 107-111, in the clean copy of the revised manuscript)**

References:

2	Avina-Zubieta, J. A., Thomas, J., Sadatsafavi, M., Lehman, A. J. & Lacaille, D. Risk of incident cardiovascular events in patients with rheumatoid arthritis: a meta-analysis of observational studies. Annals of the rheumatic diseases 71, 1524-1529, doi:10.1136/annrheumdis-2011-200726 (2012).

3	Kitas, G. D. & Gabriel, S. E. Cardiovascular disease in rheumatoid arthritis: state of the art and future perspectives. Annals of the rheumatic diseases 70, 8-14, doi:10.1136/ard.2010.142133 (2011).

4	Holmqvist, M. E. et al. Risk of venous thromboembolism in patients with rheumatoid arthritis and association with disease duration and hospitalization. Jama 308, 1350-1356, doi:10.1001/2012.jama.11741 (2012).

5	Zoller, B., Li, X., Sundquist, J. & Sundquist, K. Risk of pulmonary embolism in patients with autoimmune disorders: a nationwide follow-up study from Sweden. Lancet 379, 244-249, doi:10.1016/S0140-6736(11)61306-8 (2012).

6	Holmqvist, M., Ljung, L. & Askling, J. Mortality following new-onset Rheumatoid Arthritis: has modern Rheumatology had an impact? Ann Rheum Dis 77, 85-91, doi:10.1136/annrheumdis-2017-212131 (2018).

7	Solomon, D. H. et al. Cardiovascular morbidity and mortality in women diagnosed with rheumatoid arthritis. Circulation 107, 1303-1307, doi:10.1161/01.cir.0000054612.26458.b2 (2003).

8	Peters, M. J. et al. Does rheumatoid arthritis equal diabetes mellitus as an independent risk factor for cardiovascular disease? A prospective study. Arthritis Rheum 61, 1571-1579, doi:10.1002/art.24836 (2009).

9	Liang, K. P. et al. Incidence of noncardiac vascular disease in rheumatoid arthritis and relationship to extraarticular disease manifestations. Arthritis Rheum 54, 642-648, doi:10.1002/art.21628 (2006).

29	Bartoloni, E. et al. Immunomodulation for accelerated atherosclerosis in rheumatoid arthritis and systemic lupus erythematosus. Autoimmunity reviews 24, 103760, doi:10.1016/j.autrev.2025.103760 (2025).

30	de Araujo, L. C. T., Westerholt, A., Sandiford, A. N., Gursche, A. & Kendoff, D. Periprosthetic joint infections in patients with rheumatoid arthritis are associated with higher complication and mortality rates. Arch Orthop Trauma Surg 144, 5101-5109, doi:10.1007/s00402-024-05248-y (2024).

31	Yeganeh, M. H., Kheir, M. M., Shahi, A. & Parvizi, J. Rheumatoid Arthritis, Disease Modifying Agents, and Periprosthetic Joint Infection: What Does a Joint Surgeon Need to Know? The Journal of arthroplasty 33, 1258-1264, doi:10.1016/j.arth.2017.11.031 (2018).

**Comment 6**

Methods

How was determined the quality of data(metadata)?

**Response:** We sincerely appreciate the reviewer’s comment. The quality and reliability of the metadata in our study are underpinned by the source of our data, which is the publicly available IQVIA Medical Research Data (IMRD) database. IMRD is an electronic medical record database of records of general practitioners (GPs) in the UK and represents the UK population in demographics and medical conditions. To characterize the data quality, we followed the common methodology in the field by referencing previous validation studies of the THIN/IMRD database and benchmarking its characteristics against other UK national databases. This process is a standard approach for establishing external validity ^1-2^. Meanwhile, its high quality is demonstrated by the large body of high-impact literature that has successfully utilized this dataset to produce robust and reproducible findings.

References:

1.	Zeng C, Bennell K, Yang Z, Nguyen UDT, Lu N, Wei J, et al. Risk of venous thromboembolism in knee, hip and hand osteoarthritis: a general population-based cohort study. Annals of the rheumatic diseases. 2020;79(12):1616-24. Epub 2020/09/18. doi: 10.1136/annrheumdis-2020-217782. PubMed PMID: 32938637; PubMed Central PMCID: PMCPMC7677492.

2.	Zeng C, Dubreuil M, LaRochelle MR, Lu N, Wei J, Choi HK, et al. Association of Tramadol With All-Cause Mortality Among Patients With Osteoarthritis. Jama. 2019;321(10):969-82. Epub 2019/03/13. doi: 10.1001/jama.2019.1347. PubMed PMID: 30860559; PubMed Central PMCID: PMCPMC6439672.

**Action:** “Read codes for RA have been previously verified in the UK General Practice Research Database, with a positive predictive value (PPV) of around 80%. Approximately 60% of subjects in the UK General Practice Research Database overlap with IMRD ^39,40^.” **(Page 5, line 107-111, in the clean copy of the revised manuscript)**

References:

39	Rodriguez, L. A., Tolosa, L. B., Ruigomez, A., Johansson, S. & Wallander, M. A. Rheumatoid arthritis in UK primary care: incidence and prior morbidity. Scand J Rheumatol 38, 173-177, doi:10.1080/03009740802448825 (2009).

40	Watson, D. J., Rhodes, T., Cai, B. & Guess, H. A. Lower risk of thromboembolic cardiovascular events with naproxen among patients with rheumatoid arthritis. Arch Intern Med 162, 1105-1110, doi:10.1001/archinte.162.10.1105 (2002).

**Comment 7** How was determined the diagnosis for CVD and VTE?

**Response:** We appreciate the reviewer’s suggestion. In IMRD/THIN, clinical information is captured using the UK Read code clinical terminology—a hierarchical, NHS-standard system used to encode diagnoses, symptoms, procedures and test results at the point of care. The Read classification system is used to code specific diagnoses for CVD and VTE ^1^. Previous study has demonstrated the validity of the IMRD database in clinical and epidemiological research ^2^. We have detailed how CVD and VTE are diagnosed in the **Methods** section.

References:

1	Chisholm, J. The Read clinical classification. BMJ 300, 1092, doi:10.1136/bmj.300.6732.1092 (1990).

2	Lewis, J. D., Schinnar, R., Bilker, W. B., Wang, X. & Strom, B. L. Validation studies of the health improvement network (THIN) database for pharmacoepidemiology research. Pharmacoepidemiol Drug Saf 16, 393-401, doi:10.1002/pds.1335 (2007).

**Action:** “The secondary outcomes were incident CVD and incident VTE. A patient was considered to have had an incident CVD event at the first recording of any Read term synonymous with myocardial infarction, stroke or heart failure ^48,49^. A patient was considered to be a VTE case when he or she had a recorded code of pulmonary embolism or deep vein thrombosis along with either receiving anticoagulant therapy or a fatal outcome within one month of diagnosis ^50,51^.” **(Page 9, line 190-195, in the clean copy of the revised manuscript)**

References:

48	Garcia Rodriguez, L. A., Varas-Lorenzo, C., Maguire, A. & Gonzalez-Perez, A. Nonsteroidal antiinflammatory drugs and the risk of myocardial infarction in the general population. Circulation 109, 3000-3006, doi:10.1161/01.CIR.0000132491.96623.04 (2004).

49	Ruigomez, A., Martin-Merino, E. & Rodriguez, L. A. Validation of ischemic cerebrovascular diagnoses in the health improvement network (THIN). Pharmacoepidemiol Drug Saf 19, 579-585, doi:10.1002/pds.1919 (2010).

50	Zeng, C. et al. Risk of venous thromboembolism in knee, hip and hand osteoarthritis: a general population-based cohort study. Ann Rheum Dis 79, 1616-1624, doi:10.1136/annrheumdis-2020-217782 (2020).

51	Ogdie, A. et al. Risk of venous thromboembolism in patients with psoriatic arthritis, psoriasis and rheumatoid arthritis: a general population-based cohort study. Eur Heart J 39, 3608-3614, doi:10.1093/eurheartj/ehx145 (2018).

**Comment 8**

Results

It is lacking information about arthroplasty (tourniquet time, surgical time). How the intraoperative factors could be correlated with CVD and VTE?

**Response:** Many thanks for the reviewer’s comment. IMRD is an electronic medical record database of records of general practitioners (GPs) in the UK and represents the UK population in demographics and medical conditions. This type of database does not provide detailed records of specific surgical information. The National Institute for Health and Care Excellence (NICE) in the UK (https://www.nice.org.uk/), British Orthopaedic Association (https://www.boa.ac.uk/) and Royal College of Surgeons (https://www.rcseng.ac.uk/) have released a series of guidelines regarding joint replacement surgeries. The National Joint Registry (https://www.njrcentre.org.uk/) has also collected data related to joint replacement surgeries to provide feedback on surgical outcomes and quality. The standards for these types of surgeries are relatively uniform, which helps to reduce the bias caused by these factors on the results of our study. We have added a clarification in the Discussion section of the manuscript to acknowledge this limitation.

**Action:** “Furthermore, due to the limitations of the IMRD database, we were unable to include data on the clinical pathways before arthroplasty, VTE prophylaxis, preoperative cardiac evaluation, surgical details (e.g., tourniquet time, surgical time, type of implants, cementation or non-cementation and use of elastic stocking), and postoperative complications such as anemia and blood transfusions, which limits the scope of our analysis.” **(Page 19, line 390-396, in the clean copy of the revised manuscript)**

**Comment 9** The correlation about medications used during the surgery and CVD and VTE were not mentioned. Why not?

**Response:** We extend our gratitude to the reviewer for their constructive commentary. The use of medications has been comprehensively documented in the baseline characteristics table included within the supplementary materials. IMRD often lack comprehensive information on therapeutic parameters such as period of biological disease-modifying anti-rheumatic drugs administration. We have added a clarification in the **Discussion** section of the manuscript to acknowledge this limitation.

**Action:** “Third, the current data set does not contain information on the period of biological disease-modifying anti-rheumatic drugs (DMARDs) administration. Since studies have shown that biological DMARDs use was associated a decreased risk of mortality and cardiovascular events in patients with RA ^69,70^, without adjustment of biological DMARD could also introduce potential residual confounding.” **(Page 19, line 401-406, in the clean copy of the revised manuscript)**

References:

69	Rodriguez-Rodriguez, L. et al. Treatment in rheumatoid arthritis and mortality risk in clinical practice: the role of biologic agents. Clin Exp Rheumatol 34, 1026-1032 (2016).

70	Greenberg, J. D. et al. Tumour necrosis factor antagonist use and associated risk reduction of cardiovascular events among patients with rheumatoid arthritis. Ann Rheum Dis 70, 576-582 (2011).

**Comment 10** It is lacking subgroups analysis (gender, grade of osteoarthritis, laterality, grade of BMI).

**Response:** We thank the reviewer for raising this important point. We have indeed conducted the subgroup analysis by gender, and the results are presented in **Results** section of the manuscript. IMRD is a suboptimal data source for studying gravity of RA since it lacks sufficiently detailed lab data to make inferences about gravity of disease. Consequently, we could not undertake a comprehensive analysis and report on the gravity of RA. Moreover, this type of database does not provide detailed records of specific surgical information, and due to the data accessibility issues with IMRD, the Read code for knee or hip arthroplasty could not further distinguish between unilateral and bilateral procedures. In our data analysis, BMI was included as a statistical adjustment variable. Consequently, subgroup analyses based on BMI were not performed in the present study. However, we would be happy to conduct these additional analyses if the reviewer considers them essential, and we will incorporate the findings in the revised manuscript. We have added a clarification in the **Discussion** section of the manuscript to acknowledge this limitation.

**Action:** “First, population-based studies such as those performed in IMRD often lack information on disease activity measures and disease characteristics. Although our study was restricted among subjects with RA, we were unable to assess the stage or gravity of disease between patients with and without knee or hip arthroplasty due to the constraints of the database used in this study. It is possible that patients with more severe disease have increased all-cause mortality and risk of CVD.” **(Page 18-19, line 384-390, in the clean copy of the revised manuscript)**

“Fourthly, we are unable to distinguish between unilateral and bilateral cases, and therefore cannot further explore the distinct impacts of unilateral and bilateral procedures on the outcomes.” **(Page 19, line 406-408, in the clean copy of the revised manuscript)**

**Comment 11**

Discussion

The comparison with variables like ethnicity, social status, nutrition, age were not mentioned. It could be more explored.

**Response:** We are thankful for the reviewer’s input. Per the reviewer’s comment, we have added the corresponding content to the **Discussion** section.

**Action:** “Age and BMI are the critical determinants of surgical prognosis. Although older age is generally linked to higher postoperative complications after arthroplasty, prior studies suggest age variation does not substantially influence the likelihood of undergoing joint replacement in RA, with older patients sometimes receiving surgery earlier after diagnosis ^74,75^. Obesity is another well-established risk factor for an increased burden of comorbidities. But a previous study has reported that BMI does not affect the risk of joint arthroplasty in patients with RA, which may be due to obese patients receiving more comprehensive preoperative assessments and more proactive management of complications ^76^. Ethnic minorities and individuals from lower socioeconomic backgrounds may experience disparities in access to surgery and postoperative care, potentially affecting outcomes ^77-79^. Similarly, malnutrition, common in severe RA, may further worsen complications and mortality ^80,81^.” **(Page 20-21, line 429-440, in the clean copy of the revised manuscript)**

References:

74	Burn, E. et al. Lifetime risk of knee and hip replacement following a diagnosis of RA: findings from a cohort of 13 961 patients from England. Rheumatology (Oxford) 58, 1950-1954, doi:10.1093/rheumatology/kez143 (2019).

75	Young, B. L. et al. Trends in Joint Replacement Surgery in Patients with Rheumatoid Arthritis. J Rheumatol 45, 158-164, doi:10.3899/jrheum.170001 (2018).

76	Trieb, K., Schmid, M., Stulnig, T., Huber, W. & Wanivenhaus, A. Long-term outcome of total knee replacement in patients with rheumatoid arthritis. Joint, bone, spine : revue du rhumatisme 75, 163-166, doi:10.1016/j.jbspin.2007.06.008 (2008).

77	Feder, N. M. et al. Black Patients Had Lower Utilization Rates and Higher Acute Mortalities After Total Knee Arthroplasty. The Journal of arthroplasty 40, S183-S189, doi:10.1016/j.arth.2025.05.072 (2025).

78	Liimakka, A. P. et al. Racial and Ethnic Minority Patients Have Increased Complication Risks When Undergoing Surgery While Not Meeting Clinical Guidelines. The Journal of bone and joint surgery. American volume 106, 976-983, doi:10.2106/JBJS.23.00706 (2024).

79	Weiss, R. J., Karrholm, J., Rolfson, O. & Hailer, N. P. Increased early mortality and morbidity after total hip arthroplasty in patients with socioeconomic disadvantage: a report from the Swedish Hip Arthroplasty Register. Acta Orthop 90, 264-269, doi:10.1080/17453674.2019.1598710 (2019).

80	Gee, R., Haurin, K., Saxena, A. & DiSantis, K. I. The impact of nutritional status on surgical site infection rates among total joint arthroplasty patients: A systematic review. Am J Infect Control, doi:10.1016/j.ajic.2025.08.015 (2025).

81	McCafferty, J. P. et al. Assessing Food and Nutrition Security in Patients Undergoing Total Joint Arthroplasty. The Journal of arthroplasty 40, S164-S170, doi:10.1016/j.arth.2025.05.059 (2025).

**Comment 12** The clinical relevance (CVD and VTE) associated with cost could be mentioned in this section.

**Response:** We appreciate the reviewer’s suggestion and have added the corresponding content to the **Discussion** section.

**Action:** “Although comprehensive perioperative programs require upfront resources, they may be cost-saving by preventing downstream cardiovascular and thromboembolic events ^86,87^.” **(Page 21, line 451-453, in the clean copy of the revised manuscript)**

References:

86	Alt, V. et al. The health-economic burden of hip and knee periprosthetic joint infections in Europe : a comprehensive analysis following primary arthroplasty. Bone Jt Open 6, 298-311, doi:10.1302/2633-1462.63.BJO-2024-0225.R1 (2025).

87	Nwachukwu, B. U. et al. Current status of cost utility analyses in total joint arthroplasty: a systematic review. Clinical orthopaedics and related research 473, 1815-1827, doi:10.1007/s11999-014-3964-4 (2015).

**Comment 13** Comparison with arthroplasty registries studies could be written in this section.

**Response:** Many thanks for the reviewer’s suggestion. Per the reviewer’s comment, we have added the corresponding content to the **Discussion** section.

**Action:** “Findings from the Danish Hip Arthroplasty Registry showed overall survival of primary THA in RA patients comparable to that in OA, but did not evaluate survival benefits within RA (registry study) ^58^.” **(Page 17, line 345-347, in the clean copy of the revised manuscript)**

References:

58	Rud-Sorensen, C., Pedersen, A. B., Johnsen, S. P., Riis, A. H. & Overgaard, S. Survival of primary total hip arthroplasty in rheumatoid arthritis patients. Acta Orthop 81, 60-65, doi:10.3109/17453671003685418 (2010).

**Comment 14**

Conclusion

It can shorten according with the main findings.

**Response:** We appreciate the reviewer’s comment and have shortened the **Conclusion** section.

**Action:** “In this large population-based cohort of patients with RA, knee arthroplasty was associated with a lower risk of all-cause mortality, while both knee and hip arthroplasty were associated with a higher risk of VTE. No significant associations were observed with CVD. These findings highlight potential long-term benefits and risks of joint replacement in RA, but given the observational design and possibility of residual confounding, the results should be interpreted as associations rather than causal effects. Further studies are warranted to confirm these observations and to better understand the mechanisms underlying these associations.” **(Page 22, line 464-471, in the clean copy of the revised manuscript)**

**Reviewer #3:**

**Comment 1** I find the article long, the title long, the short and long title are not aligned. Its not in clear concise english and I don't know what the key take away points are.

**Response:** Thank you very much for taking the time to review our manuscript and for providing these constructive comments. We sincerely appreciate your frank feedback, which has helped us to identify critical areas for improvement in our work. We have carefully considered all your points and have undertaken a comprehensive revision of the manuscript.

**Reviewer #4:**

**Comment 1** Page 100-102, hypothesized that knee and hip arthroplasty may be associated with lower risks of mortality, it is sudden.

**Response:** Many thanks for the reviewer’s comment. We have revised the corresponding content in the **Introduction** section to enhance the logical coherence of the manuscript and to mitigate the abruptness of the hypothesis presentation.

**Action:** “All-cause mortality is one of the most important indicators for the net risk-benefit effect of any clinical treatment regimens ^18^. Previous studies have reported that the rate of mortality after hip and knee arthroplasty surgery is up to 4.8%, with major causes including pulmonary embolism, myocardial infarction, stroke, surgical site infections, prosthetic joint infections, and complications related to anesthesia and bleeding ^19-22^. However, for patients with RA, joint arthroplasty may significantly improve physical function, reduce pain, and enhance overall quality of life. By alleviating disability and decreasing long-term systemic inflammation, such interventions could potentially contribute to a reduction in all-cause mortality among this population over time ^23,24^. Therefore, the association of either knee or hip arthroplasty with all-cause mortality in patients with RA specifically remains understudied.” **(Page 5, line 90-101, in the clean copy of the revised manuscript)**

References:

18	Prasad, V. But how many people died? Health outcomes in perspective. Cleve Clin J Med 82, 146-150, doi:10.3949/ccjm.82a.14058 (2015).

19	Ko, K. et al. Total Knee Arthroplasty: Is It Safe? A Single-Center Study of 4,124 Patients in South Korea. Clinics in orthopedic surgery 15, 935-941, doi:10.4055/cios22088 (2023).

20	Mantilla, C. B., Horlocker, T. T., Schroeder, D. R., Berry, D. J. & Brown, D. L. Frequency of myocardial infarction, pulmonary embolism, deep venous thrombosis, and death following primary hip or knee arthroplasty. Anesthesiology 96, 1140-1146, doi:10.1097/00000542-200205000-00017 (2002).

21	Parvizi, J. et al. Revision total hip arthroplasty in octogenarians. A case-control study. The Journal of bone and joint surgery. American volume 89, 2612-2618, doi:10.2106/JBJS.F.00881 (2007).

22	Pioli, G. et al. Predictors of mortality after hip fracture: results from 1-year follow-up. Aging Clin Exp Res 18, 381-387, doi:10.1007/BF03324834 (2006).

23	Madry, H. Surgical therapy in osteoarthritis. Osteoarthritis Cartilage 30, 1019-1034, doi:10.1016/j.joca.2022.01.012 (2022).

24	Pivec, R., Johnson, A. J., Mears, S. C. & Mont, M. A. Hip arthroplasty. Lancet 380, 1768-1777, doi:10.1016/S0140-6736(12)60607-2 (2012).

**Comment 2** Page 210-214, “with and without knee arthroplasty”, did the patients performed arthroplasty or not?

**Response:** We appreciate the reviewer’s suggestion. The description in the **Results** section has been revised accordingly to mitigate potential ambiguities, specifically to clarify which patients underwent arthroplasty and which did not.

**Action:** “We identified 2,387 matched pairs of subjects with RA (n=4,774; mean age 66.5 years; 25.2% men; mean BMI 28.1 kg/m^2^). This group consisted of 2,387 RA patients who had undergone knee arthroplasty and 2,387 matched RA patients without knee arthroplasty. Additionally, we identified 1,681 matched pairs of patients with RA (n=3,362; mean age 68.2 years; 25.9% men; mean BMI 26.6 kg/m^2^). This group included 1,681 RA patients who had received hip arthroplasty and 1,681 matched RA patients without hip arthroplasty.” **(Page 10, line 228-234, in the clean copy of the revised manuscript)**

**Comment 3** Table 1-3, the number of patients is different (n=4,774 for knee arthroplasty, n=3,362 for hip arthroplasty, n=4,350 for knee arthroplasty, n=2,390 for hip arthroplasty, n=4,574 for knee arthroplasty, n=3,174 for hip arthroplasty). Are they the same patients?

**Response:** We value the reviewer’s comment. The variation in sample sizes across the tables reflects the different analytical cohorts used for each outcome. The primary outcome, all-cause mortality, was analyzed using the full included study population. In contrast, the secondary outcomes—CVD and VTE—were analyzed in subcohorts derived from the full population after applying additional exclusion criteria relevant to those specific end points. Therefore, the samples presented in Tables 1–3 correspond to distinct but overlapping groups, rather than exactly the same patients across all outcomes.

**Reviewer #5:**

**Comment 1**

TITLE

The title should not include cardiovascular risk, as these findings were not statistically significant.

**Response:** We appreciate the reviewer’s comment and have modified the **Title**.

**Action:** “Reduced mortality but elevated venous thromboembolism risk following knee and hip arthroplasty in patients with rheumatoid arthritis: a general population-based cohort study.” **(Page 1, line 1-3, in the clean copy of the revised manuscript)**

**Comment 2**

ABSTRACT

The authors stated in the conclusion that “carefully selected patients with RA” may have the benefits found from the study. Since this is a population based study, the authors cannot tell if these patients were carefully selected or not.

**Response:** We appreciate the reviewer’s insightful comment. The phrase “carefully selected patients” was intended to reflect the fact that, in clinical practice, arthroplasty candidates undergo comprehensive preoperative evaluation and risk stratification—particularly regarding comorbidities and perioperative management—which may influence postoperative outcomes. Although our observational study cannot directly quantify the extent of “careful selection”, the consistency of our results across subgroups supports the robustness of the associations. More importantly, the elevated VTE risk underscores the need for stringent thromboprophylaxis, while the observed reductions in mortality and CVD risk suggest that, with appropriate patient selection and perioperative care—including cardiovascular optimization and VTE prevention—arthroplasty may offer net benefits beyond joint-specific outcomes.

**Comment 3** The abstract on the 1st page is not the same as the abstract that is on page 21 after the title page.

**Response:** We thank the reviewer for pointing out the discrepancy between the two abstracts. The brief abstract on the first page was automatically generated by the submission system and unfortunately appears incomplete. The full and complete abstract is indeed provided on page 21. We sincerely apologize for any confusion this may have caused.

**Comment 4** The key words should include hip arthroplasty and knee arthroplasty, as arthroplasty encompasses multiple body parts. I would also add VTE and CVD (written out) if possible.

**Response:** We appreciate the reviewer’s suggestion. We have revised the **Key words**.

**Action:** “**Key words**: Rheumatoid arthritis; Hip arthroplasty; Knee arthroplasty; Mortality; Cardiovascular disease; Venous thromboembolism” **(Page 3, line 62-63, in the clean copy of the revised manuscript)**

**Comment 5**

INTRODUCTION

Line 78: I would consider clarifying this sentence to state: Based on the anatomic location of their RA, patients with RA may undergo various surgical options, such as tenosynovectomy, radiosynovectomy, arthroscopic surgery, osteotomy, joint fusion, procedure for soft-tissue release, small-joint implant arthroplasty, metatarsal-head excision arthroplasty and total joint replacement.

**Response:** Many thanks for the reviewer’s suggestion and careful examination. We have revised the sentence “Based on their customized needs, patients with RA can choose from various surgical options, such as tenosynovectomy, radiosynovectomy, arthroscopic surgery, osteotomy, joint fusion, procedure for soft-tissue release, small-joint implant arthroplasty, metatarsal-head excision arthroplasty and total joint replacement” in the **Introduction** section.

**Action:** “Based on the anatomic location of their RA, patients with RA may undergo various surgical options, such as tenosynovectomy, radiosynovectomy, arthroscopic surgery, osteotomy, joint fusion, procedure for soft-tissue release, small-joint implant arthroplasty, metatarsal-head excision arthroplasty and total joint replacement ^14^.” **(Page 4, line 83-87, in the clean copy of the revised manuscript)**

References:

14	Bullock, J. et al. Rheumatoid Arthritis: A Brief Overview of the Treatment. Med Princ Pract 27, 501-507, doi:10.1159/000493390 (2018).

**Comment 6** Line 100 – Typically the hypothesis is listed after the aim of the investigation and should be moved to Line 111.

**Response:** We appreciate the reviewer’s suggestion. We have moved the hypothesis after the aim of the investigation in the **Introduction** section.

**Action:** “The aim of this investigation was to evaluate the relationship of knee arthroplasty or hip arthroplasty to all-cause mortality, risk of CVD and incident VTE among patients with RA. To address these knowledge gaps, we conducted propensity-score matched cohort studies among patients with RA to investigate the relationship of knee or hip arthroplasty with risks of all-cause mortality, CVD and VTE, respectively, while controlling for potential confounders (including age, sex, BMI, lifestyle factors, and other comorbidities). Additionally, we performed subgroup analyses (differential risks by age and sex) to explore potential residual confounding by indication. Based on these, we hypothesized that knee and hip arthroplasty may be associated with lower risks of mortality and CVD but an increased risk of VTE among patients with RA.” **(Page 6, line 117-127, in the clean copy of the revised manuscript)**

**Comment 7**

MATERIALS AND METHODS

For the IQVIA Medical Research Database, does this include data from NHS that follows patients longitudinally? What about patients who have private insurance or see physicians privately?

**Response:** We appreciate the reviewer’s comment. The IQVIA Medical Research Database (IMRD) is a longitudinal primary-care database comprising de-identified electronic health records routinely recorded by UK general practices participating in the IQVIA Medical Research Extraction Scheme. As UK general practice functions as the NHS gatekeeper for most care, IMRD provides longitudinal follow-up while patients are registered with a contributing NHS GP, including diagnoses, prescriptions, tests, referrals and other GP-managed events (https://www.iqvia.com/locations/united-kingdom/solutions/life-sciences-industry-solutions/real-world-solutions/iqvia-medical-research-data). With respect to care delivered outside the NHS (e.g., privately insured or self-pay episodes), IMRD does not systematically capture all private sector activity nor does it encode the payer/funding source. Private care is represented only insofar as information is communicated back to the patient’s NHS GP and entered into the GP record (e.g., letters from hospitals or specialists). Therefore, IMRD can reflect some private encounters via GP-recorded correspondence, but it should not be used to estimate private-sector utilization or to distinguish NHS-funded from privately funded events (https://healthdatagateway.org/dataset/1031).

**Comment 8** Why did the authors only include patients greater than 20 years old? There are younger patients with RA who undergo joint replacement. It would be even more interesting to see the mortality in these patients.

**Response:** We appreciate the reviewer’s insightful comment regarding the inclusion of younger patients with RA who undergo joint replacement. The decision to restrict our study population to individuals over 20 years of age was based on the primary objective of this study, which aimed specifically to evaluate survival benefits among adult patients. This age threshold was pre-specified in the methodological framework to maintain consistency with prior literature in this surgical context. We fully agree that investigating mortality and postoperative outcomes in younger RA patients-particularly those under 20-represents an interesting and clinically relevant research direction. Such patients may indeed exhibit distinct profiles in terms of disease severity, surgical risks, and long-term survival. However, this group was beyond the scope of the current paper. We appreciate the suggestion and believe that exploring this specific subpopulation in future studies could provide valuable insights. We are considering such a comparative analysis in our subsequent research.

**Comment 9** The authors accessed the data on July 14, 2022 – yet the timeframe for RA diagnosis was only until 2018. Why was this the case?

**Response:** Many thanks for the reviewer’s suggestions. The sample period for this study is restricted to the years 1995 through 2018. A deliberate decision was made to exclude data from 2019 onward due to the global emergence of the COVID-19 pandemic.

**Comment 10** Line 137 – A 60% overlap is not very good. What does that mean for the accuracy of diagnosis in the database?

**Response:** We appreciate the reviewer’s constructive feedback. In this context, the “~60% overlap” refers to duplication of patient/practice coverage between UK primary-care EHR sources (for example CPRD/GPRD and THIN/IMRD) and is a sampling-frame issue-relevant to deduplication when pooling datasets, not to per-database diagnostic misclassification. Studies show substantial cross-membership of patients across CPRD and THIN (e.g., >60% for specific drug initiator cohorts), and established methods/bridge files exist to address deduplication where needed. By contrast, the accuracy of RA case identification within each database has been independently validated: RA Read-code diagnoses in GPRD/CPRD show high validity against clinical confirmation, and algorithms that combine diagnostic codes with supporting evidence (for example, ≥2 RA codes, DMARD prescriptions, or rheumatology referrals) achieve positive predictive values typically in the ~80-90% range in UK primary-care EHR research. Therefore, the reported 60% overlap does not imply poor diagnostic accuracy; it simply cautions against double-counting when combining sources, while validated RA code lists and algorithms within a single database (such as IMRD) maintain strong PPV ^1-4^.

References:

1.	Carbonari DM, Saine ME, Newcomb CW, Blak B, Roy JA, Haynes K, et al. Use of demographic and pharmacy data to identify patients included within both the Clinical Practice Research Datalink (CPRD) and The Health Improvement Network (THIN). Pharmacoepidemiol Drug Saf. 2015;24(9):999-1003. Epub 2015/07/28. doi: 10.1002/pds.3844. PubMed PMID: 26213344.

2.	Chung CP, Rohan P, Krishnaswami S, McPheeters ML. A systematic review of validated methods for identifying patients with rheumatoid arthritis using administrative or claims data. Vaccine. 2013;31 Suppl 10:K41-61. Epub 2013/12/18. doi: 10.1016/j.vaccine.2013.03.075. PubMed PMID: 24331074.

3.	Scott IC, Whittle R, Bailey J, Twohig H, Hider SL, Mallen CD, et al. Rheumatoid arthritis, psoriatic arthritis, and axial spondyloarthritis epidemiology in England from 2004 to 2020: An observational study using primary care electronic health record data. Lancet Reg Health Eur. 2022;23:100519. Epub 2022/10/18. doi: 10.1016/j.lanepe.2022.100519. PubMed PMID: 36246147; PubMed Central PMCID: PMCPMC9557034.

4.	Thomas SL, Edwards CJ, Smeeth L, Cooper C, Hall AJ. How accurate are diagnoses for rheumatoid arthritis and juvenile idiopathic arthritis in the general practice research database? Arthritis Rheum. 2008;59(9):1314-21. Epub 2008/09/02. doi: 10.1002/art.24015. PubMed PMID: 18759262.

**Comment 11** What coding was used to determine hip and knee replacements? We commonly use CPT codes. Were these total hip and total knee replacements? Were partial knee replacements included? Were total hip replacements for fracture included? Patients who undergo total hip for fracture are very different than elective patients.

**Response:** We sincerely appreciate the reviewer’s valuable suggestion. The data we analysed were sourced from IQVIA Medical Research Data (IMRD). IMRD is an electronic medical record database of records of general practitioners (GPs) in the UK and represents the UK population in demographics and medical conditions. The Read classification system is used to code specific diagnoses for CVD and VTE ^1^. Previous study has demonstrated the validity of the IMRD database in clinical and epidemiological research ^2^. Read codes for knee or hip arthroplasty have been previously verified in the UK General Practice Research Database. The inclusion criterion for the analysis was solely the presence of a readcode for joint arthroplasty. No further stratification was performed regarding the type of procedure (e.g., total vs. partial joint replacement) or its indication (e.g., fracture vs. osteoarthritis). And a statistical adjustment was applied to account for the potential confounding effect of hip fracture.

References:

1	Chisholm, J. The Read clinical classification. BMJ 300, 1092, doi:10.1136/bmj.300.6732.1092 (1990).

2	Lewis, J. D., Schinnar, R., Bilker, W. B., Wang, X. & Strom, B. L. Validation studies of the health improvement network (THIN) database for pharmacoepidemiology research. Pharmacoepidemiol Drug Saf 16, 393-401, doi:10.1002/pds.1335 (2007).

**Comment 12** Is there any access to imaging records (e.g. Ultrasound) to record DVT?

**Response:** We are thankful for the reviewer’s input. IMRD is an electronic medical record database of records of general practitioners (GPs) in the UK and represents the UK population in demographics and medical conditions. The computerized information includes socio-demographics, anthropometrics, lifestyle factors, details from visits to GPs, diagnoses from specialists’ referrals and hospital admissions. And this type of database does not provide detailed imaging records (e.g. Ultrasound) to record DVT. We have added a clarification in the **Discussion** section of the manuscript to acknowledge this limitation.

**Action:** “Furthermore, due to the limitations of the IMRD database, we were unable to include data on the clinical pathways before arthroplasty, radiographs for the assessment of joint injury severity, VTE prophylaxis, imaging for DVT, preoperative cardiac evaluation and anticoagulation controlled, surgical details (e.g., tourniquet time, surgical time, type of implants, cementation or non-cementation and use of elastic stocking), and postoperative complications such as anemia and blood transfusions, which limits the scope of our analysis.” **(Page 19, line 390-396, in the clean copy of the revised manuscript)**

**Comment 13** For VTE in the patient population, the findings may be skewed because these are patients in the UK only. How can risk factors for thromboembolism (e.g. Factor V Leiden, etc) affect patient outcomes? Was preoperative anticoagulation controlled for in the analysis?

**Response:** We thank the reviewer for this insightful comment. Information on genetic predispositions to thromboembolism (such as Factor V Leiden) and preoperative anticoagulation is not routinely captured in the IMRD database, and therefore these potential confounders could not be fully accounted for in our analysis. We have added a statement in the **Discussion** section to acknowledge this as a limitation of the study.

**Action:** “Furthermore, due to the limitations of the IMRD database, we were unable to include data on the clinical pathways before arthroplasty, radiographs for the assessment of joint injury severity, VTE prophylaxis, imaging for DVT, preoperative cardiac evaluation and anticoagulation controlled, surgical details (e.g., tourniquet time, surgical time, type of implants, cementation or non-cementation and use of elastic stocking), and postoperative complications such as anemia and blood transfusions, which limits the scope of our analysis.” **(Page 19, line 390-396, in the clean copy of the revised manuscript)**

**Comment 14** How do you define the mean time for follow-up for patients who did not undergo hip/knee arthroplasty? What was the timepoint that was defined as the start time? Many people are diagnosed with RA at much younger ages, so the follow up of approximately 5.5 years seems short for the non-arthroplasty group.

**Response:** We thank the reviewer for this important comment. In our study, the start of follow-up (index date) was defined as the date of knee or hip arthroplasty for surgical patients. For non-arthroplasty comparators, we assigned a randomly selected “pseudo-surgery date” within the same accrual block, matched to the distribution of surgery dates in the arthroplasty group. This approach ensured temporal comparability between groups and avoided immortal time bias. Consequently, the mean follow-up of approximately 5.5 years represents the period after this index date rather than from the time of RA diagnosis. We acknowledge that this design does not capture outcomes from the onset of RA, which typically occurs at a younger age, and we have clarified this point in the **Methods** section.

**Action:** “The covariate assessment period spanned two years prior to index date (defined as the date of knee or hip arthroplasty for surgical subjects and a randomly assigned “pseudo-surgery date” within the same cohort accrual block for non-surgical comparators) for medications and healthcare usage, the most recent visit prior to the index date for sociodemographic and lifestyle factors, and any time before the index date for comorbidities ^34,42-45^. This design ensured that both groups were aligned in calendar time and disease course, and that follow-up was measured consistently from a comparable index date rather than from the time of RA diagnosis.” **(Page 8, line 171-178, in the clean copy of the revised manuscript)**

References:

34	Ozen, G., Pedro, S., Schumacher, R., Simon, T. & Michaud, K. Risk factors for venous thromboembolism and atherosclerotic cardiovascular disease: do they differ in patients with rheumatoid arthritis? RMD Open 7, doi:10.1136/rmdopen-2021-001618 (2021).

42	Kim, S. C., Schneeweiss, S., Liu, J. & Solomon, D. H. Risk of venous thromboembolism in patients with rheumatoid arthritis. Arthritis care & research 65, 1600-1607, doi:10.1002/acr.22039 (2013).

43	Li, H. et al. Risk of COVID-19 Among Unvaccinated and Vaccinated Patients With Rheumatoid Arthritis: A General Population Study. Arthritis care & research 75, 956-966, doi:10.1002/acr.25028 (2023).

44	Singh, J. A. & Lewallen, D. G. Ninety-day mortality in patients undergoing elective total hip or total knee arthroplasty. The Journal of arthroplasty 27, 1417-1422 e1411, doi:10.1016/j.arth.2012.03.008 (2012).

45	Turkiewicz, A., Neogi, T., Bjork, J., Peat, G. & Englund, M. All-cause Mortality in Knee and Hip Osteoarthritis and Rheumatoid Arthritis. Epidemiology 27, 479-485, doi:10.1097/EDE.0000000000000477 (2016).

**Comment 15** KA and HA are not commonly used terms – it is typically total knee arthroplasty (TKA) and total hip arthroplasty (THA)

**Response:** Many thanks for the reviewer’s suggestions. In our data processing, all study subjects with a readcode indicating joint arthroplasty were included in the analysis, without distinguishing whether the procedure was total joint arthroplasty. Currently, there is no available information to confirm that the included subjects underwent total joint arthroplasty. Therefore, to avoid potential misunderstanding, we have retained the original terminology, namely HA and KA.

**Comment 16**

DISCUSSION

I would avoid words like “pioneering” – line 302

**Response:** We are thankful for the reviewer’s feedback and have revised the wording in the **Discussion** section.

**Action:** “In this general practice cohort representative of the UK population, our findings showed a substantially lower risk of all-cause mortality (23% for KA, 13% for HA) in patients with RA after knee or hip arthroplasty, indicating significant protective associations for both KA and HA regarding all-cause mortality. Our study revealed that both KA and HA demonstrated cardioprotective properties in patients with RA, exhibiting a trend toward reduced cardiovascular disease risk (14% for KA, 16% for HA). However, this potential cardiovascular benefit must be weighed against a substantial elevation in VTE risk, with postoperative analyses showing 63% and 119% increased incidence rates for KA and HA recipients respectively.” **(Page 16, line 323-331, in the clean copy of the revised manuscript)**

**Comment 17** The discussion is very long and I would recommend reducing the volume of words.

**Response:** We appreciate the reviewer’s suggestion. In accordance with your recommendations, revisions have been made to shorten and refine the **Discussion** section.

**Action:** Revised the **Discussion** section. **(Page 16-21, line 323-461, in the clean copy of the revised manuscript)**

**Comment 18** Limitations – should add that there are no radiographs, so there is no ability to assess severity of the joint prior to surgery.

**Response:** We acknowledge and appreciate the reviewer’s observation. In accordance with the reviewer’s recommendation, we have added the corresponding content to the **Discussion** section.

**Action:** “Furthermore, due to the limitations of the IMRD database, we were unable to include data on the clinical pathways before arthroplasty, radiographs for the assessment of joint injury severity, VTE prophylaxis, imaging for DVT, preoperative cardiac evaluation and anticoagulation controlled, surgical details (e.g., tourniquet time, surgical time, type of implants, cementation or non-cementation and use of elastic stocking), and postoperative complications such as anemia and blood transfusions, which limits the scope of our analysis.” **(Page 19, line 390-396, in the clean copy of the revised manuscript)**

**Comment 19**

CONCLUSION

The conclusion is problematic. First, I would avoid writing terms like “first evidence”. Secondly, the authors are hinting that patients with RA should get a joint replacement to reduce mortality. I think that’s worrisome as some patients may ask for a joint replacement to improve longevity, when it’s not a causal effect and it’s just correlation. Third, how do we know that these patients were carefully selected? It is impossible to tell in these population studies.

**Response:** We thank the reviewer for these valuable comments. We agree that our findings should not be interpreted as causal, nor should they be taken to imply that joint replacement should be performed to reduce mortality in patients with RA. We have revised the **Conclusion** to avoid terms such as “first evidence” and to clearly state that our results represent observational associations. We have also acknowledged the possibility of residual confounding due to patient selection, which is inherent in population-based studies, and emphasized the need for future research to validate these findings.

**Action:** “In this large population-based cohort of patients with RA, knee arthroplasty was associated with a lower risk of all-cause mortality, while both knee and hip arthroplasty were associated with a higher risk of VTE. No significant associations were observed with CVD. These findings highlight potential long-term benefits and risks of joint replacement in RA, but given the observational design and possibility of residual confounding, the results should be interpreted as associations rather than causal effects. Further studies are warranted to confirm these observations and to better understand the mechanisms underlying these associations.” **(Page 22, line 464-471, in the clean copy of the revised manuscript)**
