# Supplementary material for: Reduced mortality but elevated venous thromboembolism risk following knee and hip arthroplasty in patients with rheumatoid arthritis: A general population-based cohort study
Source: PLoS One. 2025 Nov 7;20(11):e0335453. doi: 10.1371/journal.pone.0335453 (PMC12594356; doi:10.1371/journal.pone.0335453)
Supplement: Attachment — Submitted filename: Plos one reviewer comments.docx [file pone.0335453.s004.docx]

**Plos one reviewer comments**

1. A title of 27 words is very long; please cut it down.
2. The full title and the short title are not similar or resemble each other
3. Was this article sent to an English editor, as I see several grammatical errors?

**Abstract**

Total hip and knee arthroplasty is indicated for patients with rheumatoid arthritis (RA) who experiencesignificant joint pain and have joint deformities. damage, ,

However, studies on its survival benefits among patients with RA are limited.

Scarce. was.We aimed to assess the relationship of knee and hip arthroplasty to

all-cause mortality, relative risk of cardiovascular disease (CVD), and incident venous

thromboembolism (VTE) among patients with RA. We included patients with RA

(ages≥20 years) from large and extensive United Kingdom primary care database (i.e., IQVIA

Medical Research Database). The primary outcome was all-cause mortality (n=4,774

for knee arthroplasty (n=3,362 for hip arthroplasty). The secondary outcomes included

incident CVD (n=4,350 for knee arthroplasty, n=2,390 for hip arthroplasty) and incident

VTE (n=4,574 for knee arthroplasty, n=3,174 for hip arthroplasty). We conducted

propensity score-matched cohort studies to compare the risks of each outcome

between subjects with and without knee arthroplasty (n=2,387 each) and those with

and without hip arthroplasty (n=1,681 each), respectively. We found that subjects with

knee arthroplasty had a 23% lower mortality than those without knee

arthroplasty (HR: 0.77, 95%CI: 0.65-0.90). Similarly, a lower, albeit non-statistically

A significant mortality riskwas observed among subjects with hip arthroplasty compared to

those without arthroplasty (HR: 0.87, 95%CI: 0.73-1.04). Compared with those without

arthroplasty, subjects with knee or hip arthroplasty had a lower risk of CVD. The

corresponding HRs were 0.86 (95%CI: 0.73-1.01) and 0.84 (95%CI: 0.69-1.02),

respectively. Both subjects with knee or hip arthroplasty showed a higher risk of VTE

than their counterparts (HR for knee arthroplasty: 1.63 [95%CI: 1.23-2.17]; HR for hip

arthroplasty: 2.19 [95%CI: 1.54-3.11]). The associations of arthroplasty with the risks of

mortality, CVD, and VTE were generally consistent across strata of age and sex, with

HR ranges from 0.71 to 3.75 for knee arthroplasty

**Strengths and limitations**

I found this section too long; please choose two strengths and two limitations. This section should be a maximum of 2 paragraphs.

**Conclusion**

The conclusion is tangled and I am not sure what the authors are trying to say.

**While arthroplasty can help with joint function, the authors suggest that, in certain carefully selected RA patients, it could also have survival benefits and potential cardiovascular advantages. However, there’s also a downside—a higher risk of blood clots (VTE).**
